# Natural variation in the atypical resistance gene *Ptr* confers broad‐spectrum neck blast resistance in rice

**DOI:** 10.1002/tpg2.70290

**Published:** 2026-08-12

**Authors:** Ian Paul Navea, Mohammad Abdul Monsur, Mary Jeanie Telebanco‐Yanoria, Dianne Gene De La Rosa, Sherry Lou Hechanova, Arvin Paul Tuaño, Christian Joseph Cumagun, Il‐Ryong Choi, Suresh Kadaru, Sung‐Ryul Kim, Bo Zhou, Van Schepler‐Luu

**Affiliations:** ^1^ Rice Breeding Innovation Department The International Rice Research Institute Laguna The Philippines; ^2^ Bangladesh Rice Research Institute Gazipur Bangladesh; ^3^ Institute of Chemistry, College of Arts and Sciences University of the Philippines Los Baños Laguna The Philippines; ^4^ Institute of Weed Science, Entomology, and Plant Pathology, College of Agriculture and Food Science University of the Philippines Los Baños Laguna The Philippines

## Abstract

Neck blast (NB), caused by *Magnaporthe oryzae*, infects rice (*Oryza sativa* L.) and reduces yield. Knowledge of NB resistance remains limited due to the lack of reliable resistance evaluation methods. Here, we applied a newly established neck injection method and performed a genome‐wide association study (GWAS) on 335 diverse accessions from the 3K Rice Genomes Project to identify loci associated with NB resistance. We detected a significant association on chromosome 12 (*qNB1*) explaining 15%–18% of the phenotypic variations caused by a highly virulent blast isolate (M64‐1‐3‐9‐1). Linkage disequilibrium analysis refined this region to a 42.3‐kb interval containing *Ptr*, a known gene for leaf blast resistance. We found that two *Ptr* allelic variants, *Ptr_a_* and *Ptr_c_*, both harboring the variant A/G (Lys879) in the last exon (Chr12:10,833,400), are associated with NB resistance. IR64 and the CO39 near‐isogenic line (NIL) (IRBLta2‐Pi[CO]), both harboring *Ptr_a_*, were resistant, whereas CRISPR‐Cas9 knockout of *Ptr_a_* in IR64 caused susceptibility to M64‐1‐3‐9‐1 and IK81‐25 isolates. Furthermore, the CO39 near‐isogenic line (IRBLta2‐Pi[CO]) and Lijiangxintuanheigu monogenic line (IRBLta2‐Pi) harboring the *Ptr_a_* allele exhibited resistance to 75% (six of eight isolates) and 85% (17 of 20 isolates) of Philippine differential blast isolates, respectively. Haplotype analysis revealed a superior haplotype containing two major single‐nucleotide polymorphism (SNPs; A/G and A/C at Chr12:10,833,400 and Chr12:10,845,095, respectively) and occurring in 83% of International Rice Research Institute (IRRI) elite *indica* breeding lines. These SNPs can be used to select NB‐resistant genotypes with an accuracy of 86%. Our findings identify *Ptr_a_* as a major contributor to NB resistance and provide a valuable genetic resource for developing blast‐resistant rice.

## Plain Language Summary

Rice blast, caused by the fungus *Magnaporthe oryzae*, is a major threat to global rice production. Neck blast (NB) is the most severe type of blast. However, the genetic basis of NB resistance remains poorly understood. Here, we artificially inoculated 335 rice accessions with a highly virulent NB isolate and conducted a genome‐wide association study to identify genes underlying the resistance. We identified a major locus, *qNB1*, spanning a 2.5‐Mb region on chromosome 12 associated with NB resistance. Within this locus, two allelic variants of the *Ptr* gene *(Ptr_a_* and *Ptr_c_*) were associated with NB resistance. Knockout of the *Ptr_a_* allele made a resistant rice variety susceptible while introgression into two susceptible rice varieties enhanced resistance to 17 Philippine NB isolates, confirming its role in NB resistance. A superior haplotype defined by two SNPs (A/G and A/C at Chr12:10,833,400 and Chr12:10,845,095, respectively) is present in 83% of the International Rice Research Institute's (IRRI) elite breeding lines and can be used to select NB‐resistant genotypes with an accuracy of 86%. These findings shed light on the genetic basis of NB resistance and provide breeders with practical genetic markers to identify and develop NB‐resistant rice varieties.

**Abbreviations**
3K‐RGP: 3000 Rice Genomes Project
ANOVA: analysis of variance
*AVR*
: avirulence (effector)
BC: backcross generation
BC_1_
: first backcross generation
BC_6_
: sixth backcross generation
Cas9: CRISPR‐associated protein9
CRD: completely randomized design
CRISPR: clustered regularly interspaced short palindromic repeats
DAS: days after sowing
DI: disease incidence
DMRT: Duncan's multiple range test
dpi: days post inoculation
DSI: disease severity index
gRNA: guide RNA
GWAS: genome‐wide association study
H1: superior haplotype 1
IRBL: IRRI‐bred monogenic line
IRGSP: International Rice Genome Sequencing Project
IRRI: International Rice Research Institute
LD: linkage disequilibrium
LL: lesion length
LRR: leucine‐rich repeat
LTH: Lijiangxintuanheigu
MAF: minor allele frequency
MLM: mixed linear model
MR: moderately resistant
MS: moderately susceptible
NB: neck blast
NBS‐LRR: nucleotide‐binding site‐leucine‐rich repeat
NLR: nucleotide‐binding leucine‐rich repeat
PAM: protospacer adjacent motif
PCA: principal component analysis
PCR: polymerase chain reaction
qPCR: quantitative PCR
qRT‐PCR: quantitative reverse transcription PCR
QTL: quantitative trait locus
SNP: single‐nucleotide polymorphism

## 1 INTRODUCTION

Rice blast, caused by *Magnaporthe oryzae*, represents one of the most serious constraints to the production of rice (*Oryza sativa* L.) (H. Zhang et al., 2016). The disease is responsible for up to 30% of global rice losses, enough to feed 60 million people (Nalley et al., 2016). There are several types of rice blast, including leaf blast, node blast, neck blast (NB), and panicle blast, depending on where the pathogen attacks. Leaf and NB are the most common forms of the disease, with the pathogen typically infecting leaves during the vegetative stage and the neck during the reproductive and ripening stages. Neck infection, regarded as the most damaging form, causes necrosis, interferes with nutrient assimilation and water movement, leads to panicle breakage, and results in either sterile panicles when infection occurs before the milk stage or poorly filled grains when it occurs at later stages (Kato, 2001).

Over 100 leaf blast resistance genes have been identified. However, it is unclear whether these genes can also provide resistance against NB. Nineteen leaf blast resistance genes encode nucleotide‐binding site leucine‐rich repeat (NBS‐LRR) proteins that recognize pathogen effectors in a gene‐for‐gene manner and trigger downstream immune responses, including the induction of pathogenesis‐related (PR) genes (Cesari et al., 2013; Okuyama et al., 2011; Sharma et al., 2012). Other genes, such as *Ptr*, *pi21*, *Pi‐d2*, and *Bsr‐d1*, encode non‐NBS‐LRR proteins that can activate defense pathways, including PR genes such as *OsPR1a*, *OsPR1b*, and *OsWRKY45* (X. Chen et al., 2006; Zhao et al., 2018; Zhou et al., 2022). Among the leaf blast resistance genes, *Ptr* (also known as *Pi‐ta2*, LOC_Os12g18729) confers broad‐spectrum resistance to leaf blast (Greenwood et al., 2024; G. Xiao et al., 2024). Previously, *Ptr* was mapped near *Pi‐ta* and *Pi‐ta2* and was identified as a specific allele of the armadillo‐repeat protein encoded by LOC_Os12g18729 (Zhao et al., 2018). Subsequent work showed that *Pi‐ta2* and *Ptr* are allelic and encode an identical protein (Meng et al., 2020). Whether *Ptr/Pi‐ta2* can contribute to NB resistance has not been investigated.

NB progresses into panicle blast over time. Field screening has identified several QTLs associated with panicle blast, including *qPBR10‐1* (Y. Wu et al., 2021), *qPb6‐1* (Fang et al., 2019), *qPbh‐11‐1, qPbh‐7‐1* (Fang et al., 2016), *Pi‐jnw1* (R. Wang et al., 2016), *qPbm11* (Ishihara et al., 2014), and *qPBR1* (Jinlong et al., 2024; J. Wang et al., 2024). To date, four panicle blast resistance genes, including *Pb1*, *Pb2*, *Pb3*, and *Pb4*, have been cloned and functionally characterized. However, the effectiveness of *Pb1* depends on additional QTLs. The presence of natural resistance alleles and associated markers of *Pb2*, *Pb3*, and *Pb4* in elite varieties has not been examined. It is also unclear whether these panicle blast resistance genes can provide resistance against NB. Previous studies indicate that panicle blast activates defense pathways distinct from those activated by leaf blast. These include cell wall reinforcement and reactive oxygen species formation. Pathogen gene expression may also differ between the two tissues during infection (Du et al., 2024). Although some quantitative trait loci (QTLs) overlap, tissue‐ and stage‐specific responses suggest largely independent pathways (Kalia & Rathour, 2019).

Coupling genome‐wide association study (GWAS) for resistance gene discovery with clustered regularly interspaced short palindromic repeats (CRISPR)‐Cas9‐mediated gene editing for gene validation can fast‐track the identification of new disease resistance genes. GWAS exploits natural variation and historical recombination in diverse populations, providing high mapping resolution while reducing reliance on bi‐parental populations (Nakano & Kobayashi, 2020). The 3K‐RGP is particularly powerful because of its dense marker coverage and diverse genetic base (Mansueto et al., 2016). Using this panel, novel *Ptr* and *Pia* alleles linked to leaf blast resistance were identified (Greenwood et al., 2024). In addition, 68 loci for blast resistance (Fan et al., 2024) and multiple loci for bacterial blight (Lu et al., 2021) were detected. These studies highlight the utility of the 3K‐RGP in disease resistance trait discovery, as its size and balanced structure increase the power of mixed linear model (MLM) and multi‐locus models. Candidate genes identified through GWAS can be rapidly validated using CRISPR‐Cas9‐mediated knockout or knock‐in approaches, which allow precise functional testing of alleles in the same genetic background (K. Chen et al., 2022; Hussain et al., 2024; Thomson et al., 2022). However, identifying NB resistance genes through GWAS has not been possible due to challenges in establishing reliable and reproducible evaluation protocols on a large set of rice accessions.

Several NB inoculation methods have been described, including brushing spore suspensions, placing infected leaf fragments from *Paspalum dilatatum* or *Brachiaria mutica* on the neck, wrapping the neck with cotton soaked in spore suspension, injecting spore suspensions into the neck or sheath, using plastic rings filled with conidial suspension, spraying, and applying slices of sporulating culture directly onto the neck (Bhaskar et al., 2017; Ghatak et al., 2013; Koga et al., 2004; Korinsak et al., 2023; Singh et al., 2021). Despite these various approaches, no standard inoculation method and disease measurement scale are available, and none have been applied to screen a large set of rice accessions for QTL mapping.

Recently, we optimized the neck injection method (Yanoria et al., 2025) by covering inoculated plants with lightweight polyethylene bags for 24 h after injecting conidial suspension into the necks to favor pathogen proliferation. We also developed an NB severity scale that includes disease incidence, disease severity index, and lesion length. This enabled us to screen a large number of plants under semi‐controlled greenhouse conditions. Here, we used this newly established artificial inoculation method and NB severity scale to conduct GWAS on the 335 rice accessions from the 3K‐RGP, leading to the identification of a major NB resistance locus located on chromosome 12 harboring *Ptr* with two natural variants, *Ptr_a_* and *Ptr_c_*, highly associated with NB resistance. The role of the *Ptr_a_* allele in NB resistance was characterized via gene introgression and CRISPR‐Cas9‐mediated gene knockout. To support the deployment of *Ptr_a_*, we identified gene‐based markers and surveyed International Rice Research Institute's (IRRI) elite *indica* breeding lines for the presence of the resistance allele, linking trait discovery directly to breeding applications.

## 2 MATERIALS AND METHODS

### 2.1 Plant materials

A subset of 335 rice accessions (Table S1) from an original panel of 500 genetically diverse accessions described in a GWAS for leaf blast (Greenwood et al., 2024) was used. The panel was derived from the 3000 Rice Genomes Project (3K‐RGP) by excluding accessions carrying major blast resistance genes (*Pi2/9*, *Pik*, and *Pi3/5*) through gene‐specific marker screening, and by further stratifying accessions according to country of origin and genetic subgroup. The 335 accessions were evaluated for NB resistance. Lijiangxintuanheigu (LTH) and CO39 were included as susceptible controls.

For functional validation of the candidate gene (*Ptr_a_*), multiple genetic resources were used, including the BC_6_‐derived CO39 near‐isogenic line (NIL) (IRBLta2‐Pi[CO]) and the BC_1_‐derived LTH monogenic line (ML) (IRBLta2‐Pi), both harboring the *Ptr_a_* allele originating from the Pi No. 4 variety (Tsunematsu et al., 2000). Additionally, the CO39 NIL, IRBLta‐Ya[CO], harboring *Pi‐ta* (Telebanco‐Yanoria et al., 2010) was included to differentiate the resistance spectra of *Pi‐ta* and *Ptr_a_*. To calculate the frequency of the superior haplotype in breeding materials, 43 accessions were randomly selected from a panel of 101 IRRI *indica* elite breeding lines (Table S2) and evaluated for their NB reactions.

### 2.2 Plant growth conditions

Ten germinated seeds per accession were sown in plastic trays containing soil collected from the IRRI field station in Los Baños, Laguna, Philippines. Seventeen days after sowing (DAS), the seedlings were transplanted into individual plastic pots (23 cm height, 16 cm diameter) in the BG‐05 greenhouse at the IRRI (14.1735°N, 121.2594°E). Each pot was filled with 3.5 kg of soil and fertilized at transplanting with 3.6 g ammonium sulfate [(NH_4_)_2_SO_4_, 14‐0‐0], followed by additional applications 3 weeks later and before booting. The plants were grown under natural light at 25–32°C with regular watering. At 10–12 days after flowering, yellowing leaves were removed, and plants were transferred to a climatized room 1 day before artificial inoculation and maintained at 24–26°C and 60%–70% relative humidity under natural light.

### 2.3 Fungal isolates

Twenty differential leaf blast isolates were identified previously by evaluating their virulence spectrum on 25 MLs harboring individual *Pi* genes in LTH background (Kobayashi et al., 2007). This set of differential isolates represents the diverse virulence spectrum of the Philippines blast population. Among these, M64‐1‐3‐9‐1 was one of the most virulent isolates, causing susceptibility in approximately 50% of the lines, including those harboring *Pia*, *Pii*, *Pik*, *Pik‐p*, *Pik‐h*, *Pib*, *Pi1*, *Pi3*, *Pi7(t)*, *Pikm*, *Pi‐ta*, and *Pi11*. This isolate was therefore selected for NB evaluation of the GWAS panel and the IRRI *indica* elite breeding lines.

For functional validation experiments, multiple isolates were used. The *Ptr_a_* edited lines were inoculated with isolates M64‐1‐3‐9‐1 and IK81‐25, both carrying *AVR‐Pita* (avirulence [effector]), the cognate effector of *Ptr* (Selisana et al., 2017; G. Xiao et al., 2024). The *Ptr_a_* NIL and ML were evaluated using 8 and 20 differential blast isolates, respectively.

### 2.4 Neck and leaf blast resistance evaluation

NB resistance evaluation was performed using our newly established neck injection protocol (Yanoria et al., 2025). Inoculation was carried out 10–12 days after flowering by injecting 200 µL of conidial suspension (1 × 10^5^ conidia/ml) approximately 5 mm below the panicle neck with a 1‐mL syringe. The inoculated plants were covered with a polyethylene bag for 24 h immediately after inoculation to maintain high humidity and promote fungal growth. At 10 days post‐inoculation (dpi), disease symptoms were evaluated based on lesion length (LL, mm; longest lesion from the injection point), disease incidence (DI, %; proportion of symptomatic panicles relative to the total panicles inoculated), and disease severity index (DSI, %) calculated from severity scores (0–9 scale; Table S3) using the formula:

$$\begin{array}{ccc} \mathrm{DSI}\left(\%\right) & = & \frac{\sum \left(\text{class frequency}\times \text{severity score}\right)}{\text{Total number of plants}\times \text{maximum severity score}}\\ & & \times 100. \end{array}$$

Lesion length was further used to classify accessions into four categories: resistant (R, 0–7 mm), moderately resistant (MR, >7–30 mm), moderately susceptible (MS, >30–55 mm), and susceptible (S, >55 mm).

The GWAS panel, NILs, MLs, and IRRI elite *indica* breeding lines were evaluated once in a completely randomized design (CRD) with three plants per accession representing biological replicates. Disease symptoms were evaluated on three to five panicles per biological replicate. For the GWAS panel, DI, DSI, and LL data were collected, while for the other lines, only LL was measured.

For the gene functional validation experiment, the CRISPR‐Cas9‐mediated knockout lines were evaluated in two independent experiments conducted 3 weeks apart. The experiments were laid out in CRD with five biological replicates per genotype. For each biological replicate, LL was measured from three panicles. Phenotyping repeatability was evaluated by calculating the Pearson correlation coefficient between lesion lengths obtained from the two independent experiments. Lesion length measurements from the two experiments were strongly correlated (Pearson's *r* = 0.97, *p* < 0.001) (Figure S1), indicating high repeatability of the modified neck inoculation method. Given this high repeatability, data from the two independent experiments were pooled for subsequent analysis of disease response.

Leaf blast resistance evaluation was performed using the IRRI standard protocol (Yanoria et al., 2025). Briefly, a tray of 14‐day‐old seedlings was sprayed with 40 mL of the spore suspension (1 × 10^5^ conidia/ml) using an airbrush sprayer (0.5‐mm nozzle atomizer, 18–20 psi, oil‐free mini‐compressor). Disease severity was evaluated at 6 dpi using the modified lesion type scale (0–2, resistant; 3–5, susceptible) (Yanoria et al., 2025). Leaf blast evaluation was performed twice.

### 2.5 Association mapping

Trait distributions in the GWAS panel were evaluated for normality using the Kolmogorov–Smirnov test implemented in R (R Core Team, 2024). The Q‐Q plots assessing normal distribution were generated (Figure S2). Because DI and DSI showed deviation from normality (*D* = 0.34, *p* < 0.001 and *D* = 0.12, *p* < 0.001 for DI and DSI, respectively), the values were transformed using an arcsine square‐root transformation (Sokal & Rohlf, 1995) prior to GWAS. LL (*D* = 0.05, *p* = 0.31) approximated normal distribution and was analyzed without transformation. The accession‐level mean phenotypes were used for GWAS. Two and nine accessions did not have data for DI and DSI (Table S1), respectively; thus, they were excluded from the GWAS analysis for the corresponding traits.

Single nucleotide polymorphism (SNP) genotype data for the 335 rice accessions were obtained from the 3K‐RGP dataset (https://snp‐seek.irri.org/) and processed using PLINK v1.9 (Alexandrov et al., 2015; Purcell et al., 2007). The 404k core SNPs retrieved from SNP‐Seek were filtered to remove SNPs with minor allele frequency (MAF) of <5%. Filtering resulted in a total of 253,465 SNPs for GWAS. All genomic coordinates were annotated based on the *Nipponbare* reference genome, IRGSP‐1.0 (https://rice.uga.edu/). GWAS was conducted using the MLM (F. Zhang et al., 2021), in which SNP genotypes were treated as fixed effects, while population structure and kinship matrices were incorporated as random effects. Principal component analysis (PCA) for population structure estimation and kinship calculations was performed using TASSEL v5.0 (Bradbury et al., 2005).

Circular Manhattan plots were produced using the CMplot package in R (Yin et al., 2021). SNP density was visualized in 1‐Mb bins. The genome‐wide significance threshold was calculated using Bonferroni correction (*p* = 0.05/*n*, where *n* is the number of SNPs) resulting in a −log_10_(*p*) = 6.7. SNPs with −log_10_(*p*) values exceeding the significance threshold were identified as trait‐associated markers. The SNPs at Chr12:10,833,400 and Chr12:10,797,178 showed the highest −log_10_(*p*) values and were identified as the peak SNPs for DI, and for DSI and LL, respectively.

### 2.6 Haploblock analysis and candidate gene identification

Haploblock boundaries were defined by regions containing all significant SNPs in linkage disequilibrium (LD) with the peak SNPs. Two haploblocks containing SNPs with strong linkage (*r^2^* ≥ 0.8) with the peak SNPs (Chr12:10,833,400 and Chr12:10,797,178) were determined using the Solid Spine implemented in Haploview v4.2 (Barrett et al., 2005).

Candidate genes were then identified within these two haploblocks. *Ptr* (LOC_Os12g18729), a known leaf blast resistance gene, was found in the haploblock containing the DI‐peak SNP (Chr12:10,833,400), while no known blast resistance gene was identified in the haploblock containing the DSI and LL peak SNPs (Chr12:10,797,178). The haploblock of the DI‐peak SNP was therefore prioritized for haplotype analysis and candidate gene identification.

To identify the superior haplotype and SNP markers for marker‐assisted selection (MAS), haplotype analysis was carried out using significant SNPs located within the haploblock of the DI‐peak SNP. Haplotype analysis was carried out following a gene‐based association approach described in previous studies (X. Wang et al., 2017; Yano et al., 2016). The superior haplotype was defined as the one present in accession groups that exhibited resistance to NB across all three disease components, DI, DSI, and LL.

Since the *Ptr* gene is located within the haploblock containing the DI peak SNP and has five allelic variants (*Ptr_a_*, *Ptr_b_*, *Ptr_c_*, *Ptr_d_*, and *Ptr_e_*), we assessed the associations between these variants and NB resistance. Accession‐wise allele assignments were retrieved from the study of Greenwood et al. (2024). DI, DSI, and LL data were then analyzed across accession groups corresponding to each allele.

### 2.7 CRISPR‐Cas9 construct design and rice transformation

A single guide RNA (gRNA) targeting the third exon of *Ptr_a_* (LOC_Os12g18729) was designed using CRISPR RGEN Tools (http://www.rgenome.net/about/). The gRNA: 5′‐GGCACACCTGCAGCCTGCGC‐3′ (protospacer adjacent motif, PAM: TGG, Chr12:10,831,076) with the highest on‐target score was selected. Complementary single‐stranded oligonucleotides encoding the 20‐bp target sequence with *AarI* overhangs were synthesized as primers. The forward and reverse oligos were annealed and ligated into *AarI*‐digested pSR339 (Figure S3), where gRNA expression was driven by the rice U3 small nuclear RNA (*OsU3*) promoter. The construct carried the pZmUbi1‐SpCas9‐tNOS::pOSU3‐AarI‐LacZ‐AaRI‐sgRNA::p35S‐HPT‐T35S cassette. The final construct was introduced into *Agrobacterium tumefaciens* strain LBA4404 using the freeze‐thaw method (Weigel & Glazebrook, 2006). Rice transformation was performed in *O. sativa* ssp. *indica* cv. IR64 following an *Agrobacterium*‐mediated transformation protocol established at the IRRI (Slamet‐Loedin et al., 2014). Transformed T_0_ plants were genotyped using target‐region polymerase chain reaction (PCR) amplification and Sanger sequencing. Mutation analysis was performed using DSDecode (http://skl.scau.edu.cn/dsdecode/; Liu et al., 2015) with a cutoff signal ratio of 0.30, an anchor sequence length of 20 nt, and a degenerate sequence length of 15 nt. Of the primary T_0_ transformants from 182 independent events, 22 were selected for advancement to T_1_ based on predicted loss‐of‐function mutations, including frameshift mutations and premature termination codons. T_1_ plants were genotypically characterized, leading to the identification of five independent homozygous knockout T_1_ lines (*ko‐1 to ko‐5*). Deletions of 49, 11, 5, and 6 bp were observed in *ko‐1*, *ko‐2*, *ko‐3*, and *ko‐4*, respectively, whereas *ko‐5* exhibited a single‐nucleotide C insertion. These lines were advanced to T_2_ for phenotypic evaluation.

Cass‐OFFinder (Bae et al., 2014) was used to identify genome‐wide candidate off‐target sites, revealing a single site (Chr11:11,581,029) with three mismatches relative to the gRNA sequence GGCACACCTGACAGCagGCGtAGG (mismatches shown in lowercase). This site is not located in any annotated gene in the IR64 genome (https://riceome.hzau.edu.cn/). The closest coding gene (LOC_Os11g20080) is located at Chr11:11,583,390‐11,586,939, which is 2,361 bp downstream of the off‐target site. The predicted off‐target region was amplified using specific primers (Table S4) and sequenced to survey for possible mutations.

### 2.8 RNA extraction and qRT‐PCR analysis

To quantify the relative expression of *Ptr_a_* and pathogenesis‐related genes (*OsPR1b* and *OsPBZ1*), total RNA was extracted from neck tissues located approximately 20 mm below the last node of CO39, IRBLta2‐Pi[CO] (*Ptr_a_*), and IRBLta‐Ya[CO] (*Pi‐ta*) plants. Tissues were collected from M64‐1‐3‐9‐1 and mock‐inoculated plants at 1, 2, 3, and 5 dpi. Five biological replicates were used for each treatment and time point. RNA was extracted using TRIzol reagent (Thermo Fisher Scientific, Germany) according to the manufacturer's instructions. Complementary DNA was synthesized using the GoScript Reverse Transcriptase Kit (Promega). Quantitative real‐time PCR was performed using Luna Universal qPCR Master Mix (New England Biolabs) on a StepOne Real‐Time PCR System (Applied Biosystems) with gene‐specific primers (Table S4). Primer specificity was confirmed by melt curve analysis, which revealed single distinct peaks for the primer pairs corresponding to *Ptr_a_*, *OsPR1b*, and *OsPBZ1* (Figure S4). Gene expression levels were normalized to those of *OsActin1*, and relative expression was calculated using the 2^−ΔΔCt^ method (Livak & Schmittgen, 2001).

### 2.9 Statistical analyses

For the validation experiments, differences among genotypes, allelic variant groups, and gene expression (*Ptr_a_*, *OsPR1b*, and *OsPBZ1*) were analyzed using one‐way analysis of variance (ANOVA) implemented in the Agricolae package in R (Jebesa & Kenea, 2023), followed by Duncan's multiple range test (DMRT) for mean separation (Wanishsakpong et al., 2022). Reported *p* values were adjusted for multiple comparisons using either the Benjamini–Hochberg false discovery rate (Noble, 2009) or the Bonferroni method (Rastaghi et al., 2024). An adjusted *p* < 0.05 was considered statistically significant. Pairwise comparisons were conducted using two‐sample, two‐tailed Welch's *t*‐tests (Curtis, 2024), applied both to contrast *Ptr_a_* and *Pi‐ta* monogenic lines across isolates.

## 3 RESULTS

### 3.1 Rice accessions from distinct subpopulations differ in resistance against neck blast

To assess NB responses of 335 diverse rice accessions from the 3K Rice Genome Project (3K‐RGP), the plants were inoculated with M64‐1‐3‐9‐1, a highly virulent *M. oryzae* isolate from the Philippines. Three disease components, including DI (%), DSI, and LL (mm) were quantified.

**FIGURE 1:**
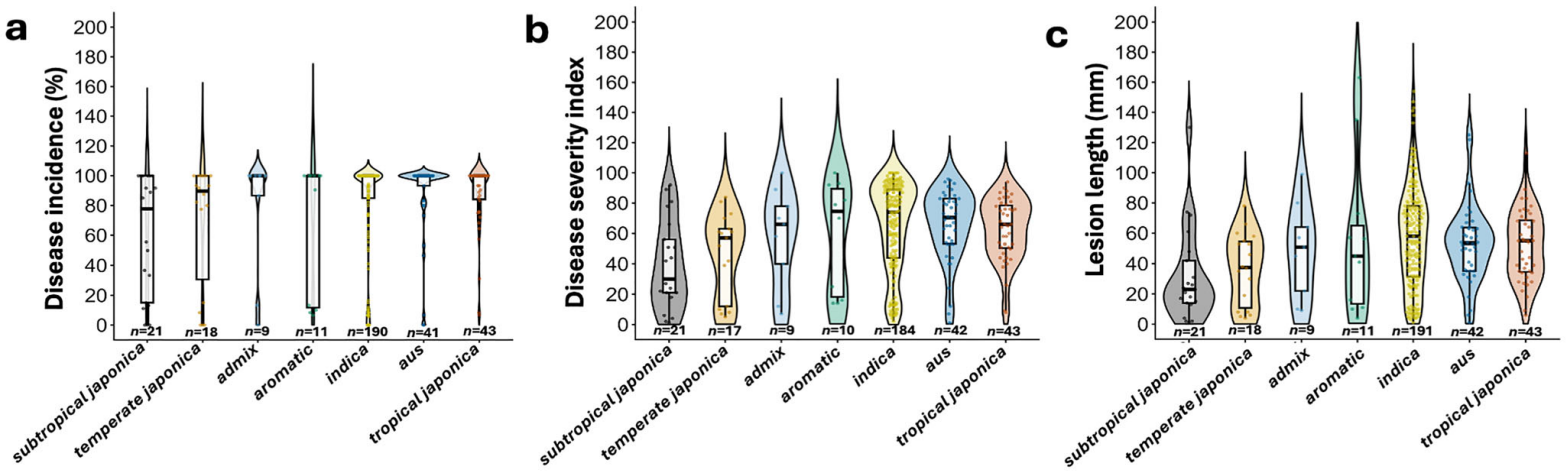
Subpopulation‐level neck blast reactions of the 335 rice accessions. (a) Disease incidence (%), (b) disease severity index, and (c) lesion length (mm). Violin plots show kernel‐density estimates; overlaid thin boxplots mark the median (line), interquartile range (IQR; box), and 1.5 × IQR whiskers; data points represent individual accessions. The number of accessions in each subpopulation is indicated by *n* below each boxplot.

The panel comprised *indica* (191), *tropical japonica* (43), *aus* (42), *subtropical japonica* (21), *temperate japonica* (18), *aromatic* (11), and *admix* (9) accessions. In total, 85 accessions were resistant (R) to MR to M64‐1‐3‐9‐1 (Table S1). We found that at the subpopulation level, *subtropical japonica* was the most resistant, showing the lowest DI, DSI, and LL values (Figure 1; Table S5). *Temperate japonica* accessions were moderately resistant, whereas the *aromatic* and *admix* groups showed intermediate resistance. The *indica* group was generally susceptible, with relatively high LL values, whereas *aus* and *tropical japonica* groups were highly susceptible, with the highest DI and DSI values. These results demonstrate subpopulation‐dependent differences in NB resistance.

### 3.2 A significant GWAS association for neck blast resistance (*qNB1*) maps to chromosome 12 and harbors the *Ptr* gene

**FIGURE 2:**
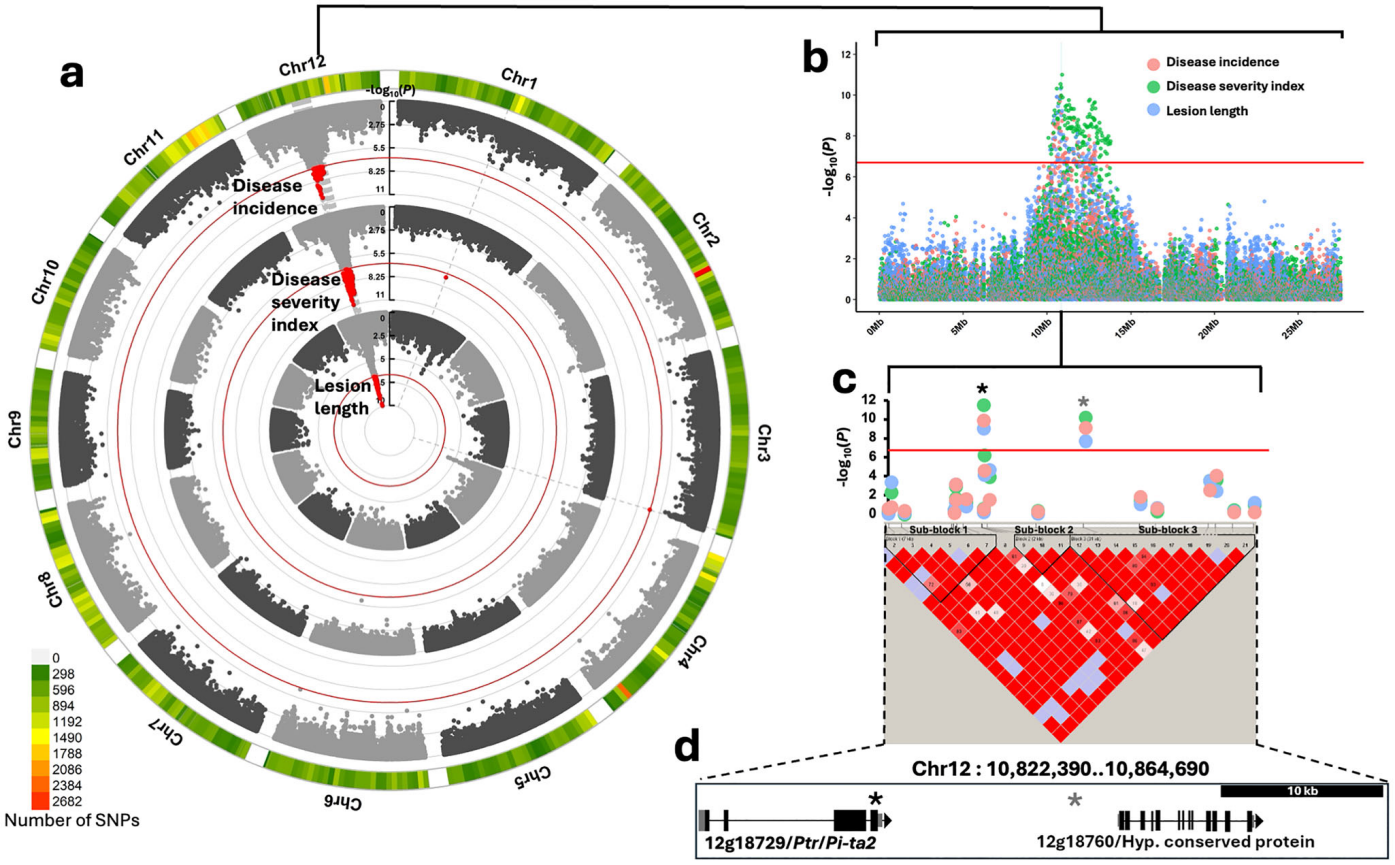
Genome‐wide association analysis of neck blast resistance in rice. (a) Circular Manhattan plot depicting associations across all 12 chromosomes for disease incidence (DI), disease severity index (DSI), and lesion length (LL). (b) Local Manhattan plot of chromosome 12 showing a major‐effect locus *qNB1* centered at a 2.5‐Mb region on Chr 12 (Chr12:10,350,016‐ Chr12:12,885,558). (c) Linkage disequilibrium (LD) heatmap of the 42.3‐kb haploblock associated with DI peak SNP (Chr12:10,833,400). (d) Close‐up view of the genomic region containing the DI haploblock. Two significantly associated SNPs with DI, DSI, and LL in DI haploblock are indicated: Chr12:10,833,400 (black asterisk) and Chr12:10,845,095 (gray asterisk). A 10‐kb scale bar is shown. Gene identifiers follow the Rice Genome Annotation Project (Osa1) Release 7 nomenclature, shortened by removing the LOC_Os prefix.

To identify loci associated with NB resistance, we performed a GWAS on 335 rice accessions for all three disease components: DI, DSI, and LL. A total of 253,465 SNPs, aligned to the *Nipponbare* reference genome, were retrieved from the SNP‐Seek database. Fifty‐nine, 126, and 60 SNPs were significantly associated with DI, DSI, and LL, respectively (Bonferroni‐corrected threshold of –log_10_(*p*) ≥ 6.7) (Table S6). These SNPs spanned a 2.5‐Mb region (Chr12:10,350,016‐Chr12:12,885,558) on chromosome 12, defining a locus hereafter named *qNB1* (Figure 2a,b). Forty‐three SNPs were significantly associated with all three disease components (DI, DSI, and LL) (Table S6). The peak SNP with the highest association with DI was an A/G SNP at Chr12:10,833,400 (–log_10_(*p*) = 9.94), whereas the peak SNP for both DSI and LL was an A/C SNP at Chr12:10,797,178 (–log_10_(*p*) = 12.22 and 10.32 for DSI and LL, respectively).

Haploblock analysis based on SNPs in linkage disequilibrium (LD) with the two peak SNPs identified two haploblocks: one associated with DI peak SNP (Chr12:10,833,400) and the other associated with DSI and LL peak SNP (Chr12:10,797,178). The haploblock associated with DSI and LL spans a 36‐kb region and does not harbor any known blast resistance genes. Meanwhile, the DI haploblock spanning a 42.3‐kb region (Chr12:10,822,390‐Chr12:10,864,690) comprised three sub‐blocks and harbored 20 significant SNPs (Figure 2c; Table S7). This DI haploblock also harbors *Ptr* (LOC_Os12g18729 or *Pi‐ta2*), a known leaf blast resistance gene, and LOC_Os12g18760, a hypothetical conserved protein (Figure 2d). We therefore used the DI haploblock for further analyses.

### 3.3 *Ptr_a_* and *Ptr_c_*, both carrying the Lys879 variant, are associated with NB resistance

**FIGURE 3:**
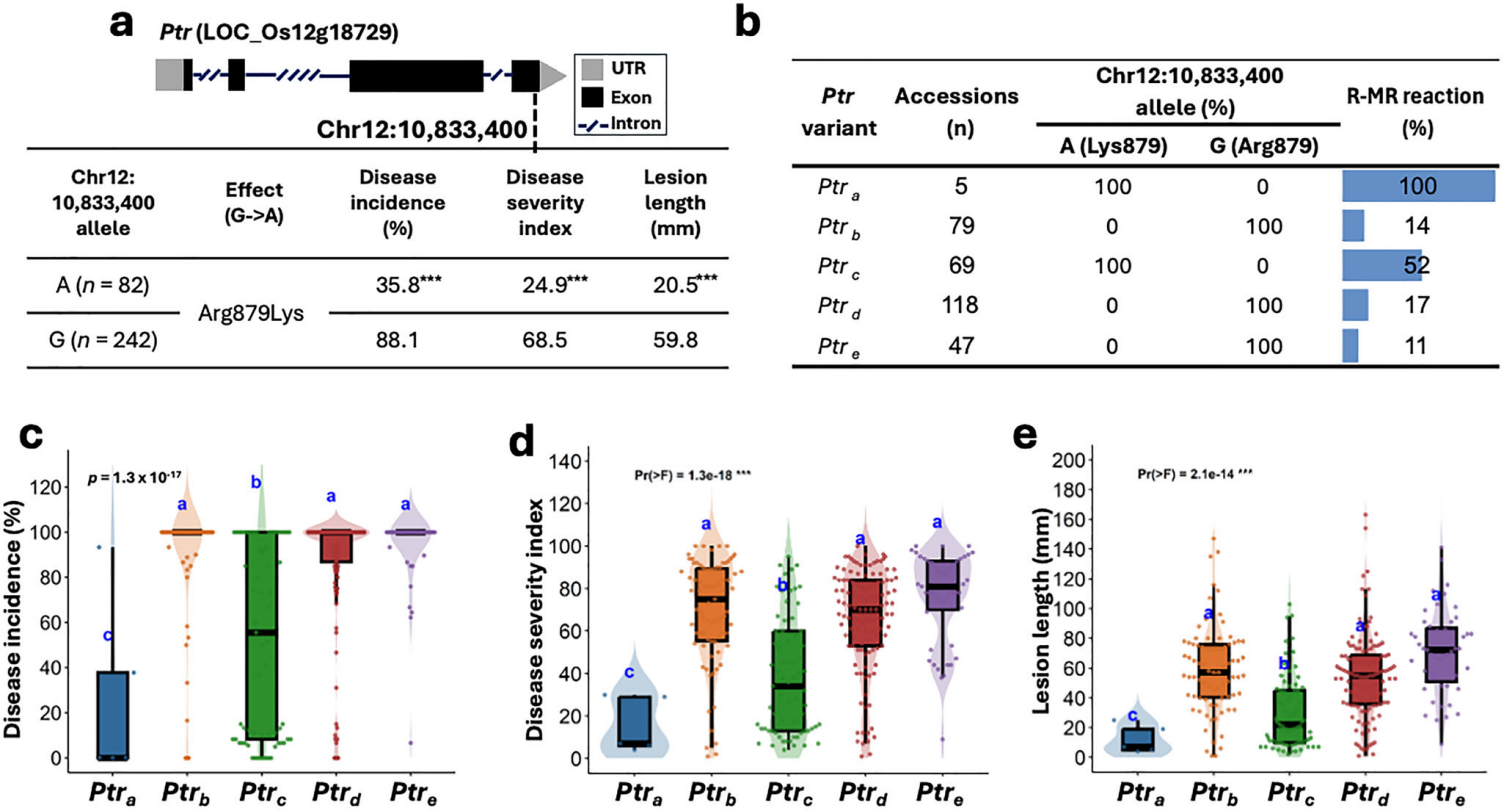
Association between neck blast resistance and allelic variation of *Ptr*. (a) Gene structure of *Ptr* (LOC_Os12g18729) showing the single nucleotide polymorphism (SNP) at Chr12:10,833,400 in the last exon. The G to A substitution results in an Arg879Lys amino acid change. The table presents percentages of disease incidence (DI), disease severity index (DSI), and lesion length (LL) in two sets of rice accessions harboring A (*n* = 82) or G (*n* = 242) allele at Chr12:10,833,400. Phenotypic differences between the two alleles were tested using a two‐tailed t‐test (***p < 0.001). (b) Distribution of the Chr12:10,833,400 alleles among the five *Ptr* variants (*Ptr_a_*–*Ptr_e_*). The percentage of resistant (R) and moderately resistant (MR) accessions within each haplotype group is indicated by the horizontal bars. Classification was based on lesion length, with accessions categorized as resistant (R, 0–7 mm) and moderately resistant (MR, >7–30 mm). (c‐e) Boxplots of DI, DSI, and LL among five *Ptr* allelic variants. ANOVA results (*p* values) are shown above each panel, and different letters above boxplots indicate significant differences among allelic groups based on Duncan's multiple range test (DMRT, *p* < 0.05).

Since the *qNB1* locus (DI haploblock) harbored *Ptr*, this gene was targeted for allelic variation analysis. *Ptr* encodes an armadillo‐repeat protein (ARM) required for *AVR‐Pita* recognition. Previous deep sequencing analysis revealed five allelic variants (*Ptr_a_‐Ptr_e_*) with polymorphisms confined to their C‐termini, specifically the last 50 residues immediately downstream of the ARM domain (Greenwood et al., 2024; G. Xiao et al., 2024). Here, the DI peak SNP at Chr12:10,833,400 introduces a nonsynonymous G‐to‐A substitution in the last exon of *Ptr* and downstream of the ARM domain, resulting in an arginine‐to‐lysine (Arg879Lys) amino acid change. To assess the functional relevance of this G/A variant, we examined its association with the NB resistance phenotype. Among the 324 accessions with available genotype data for the G/A SNP at the Chr12:10,833,400, those with the A allele (Lys879) showed significantly lower DI, DSI, and LL values than those with the G allele (Arg879) (Figure 3a).

We examined the distribution of the Chr12:10,833,400 G/A polymorphism among the five *Ptr* variants (*Ptr_a_*, *Ptr_b_*, *Ptr_c_*, *Ptr_d_*, and *Ptr_e_*). The Lys879 variant was exclusively present in accessions harboring *Ptr_a_* and *Ptr_c_*, whereas all accessions carrying *Ptr_b_*, *Ptr_d_*, and *Ptr_e_* harbored the Arg879 variant (Figure 3b; Table S8). Notably, the frequencies of resistant and moderately resistant (R‐MR) accessions were markedly higher in *Ptr_a_* (100%) and *Ptr_c_* (52%) than in *Ptr_b_* (14%), *Ptr_d_* (17%), and *Ptr_e_* (11%) (Figure 3b). Consistent with these observations, accessions harboring *Ptr_a_* or *Ptr_c_* exhibited significantly lower DI, DSI, and LL than those carrying the Arg879 variant (Figure 3c–e). Collectively, these results indicate that the Lys879‐containing *Ptr_a_* and *Ptr_c_* variants are associated with enhanced NB resistance.

### 3.4 *Ptr_a_* knockout in IR64 led to susceptibility, while its introgression into CO39 and LTH conferred resistance against a wide range of neck blast isolates from the Philippines

**FIGURE 4:**
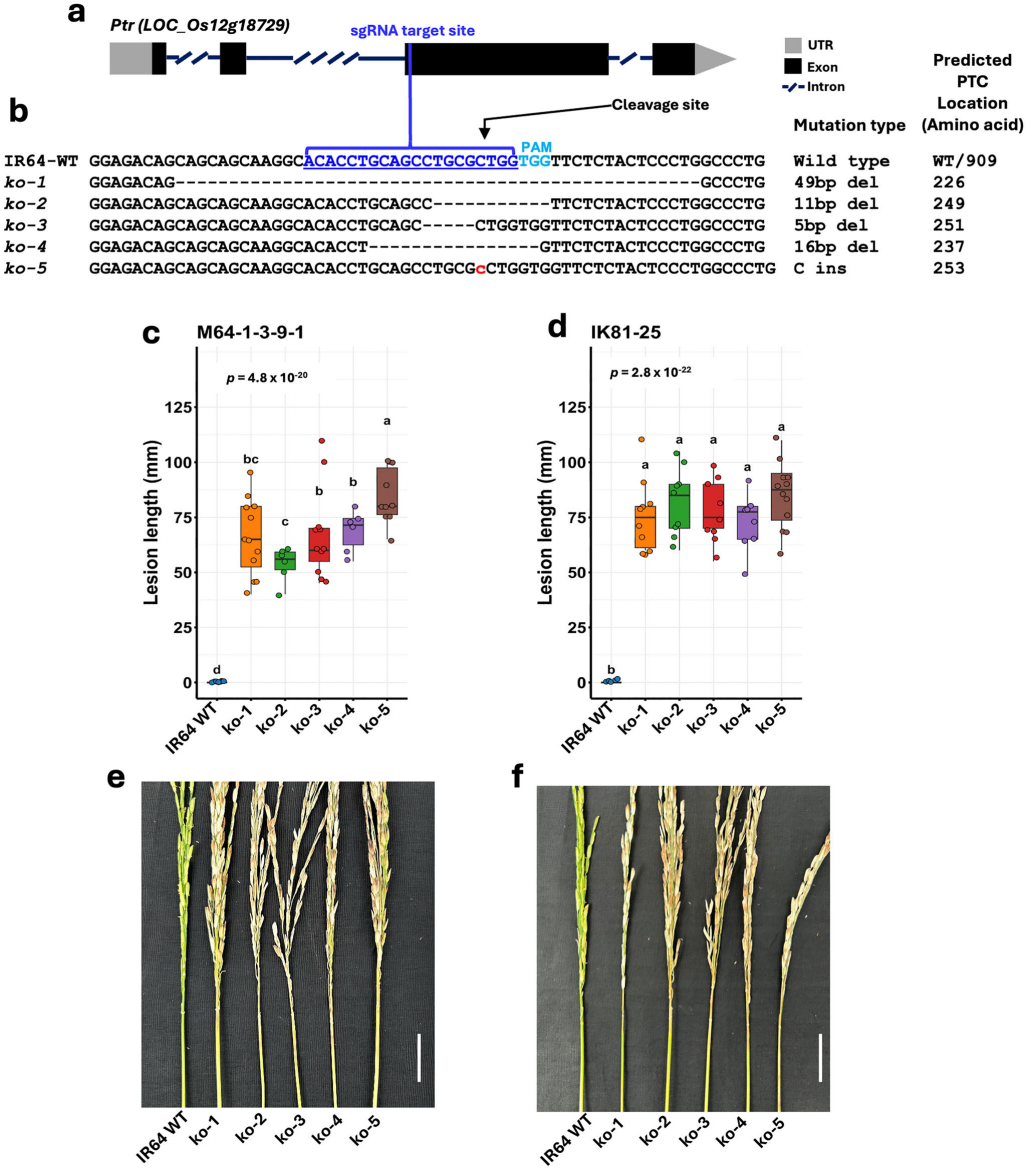
CRISPR‐Cas9‐mediated *Ptr_a_* knockout in IR64. (a) Schematic representation of gRNA target site within the genomic regions of *Ptr_a_*. (b) Mutation sites in knockout mutants generated in the IR64 background via CRISPR‐Cas9 editing. del and ins denote deletions and insertions, respectively. (c, d) Neck blast lesion lengths in knockout (*ko*) and wild‐type (WT) plants at 10 days after inoculation with M64‐1‐3‐9‐1 and IK81‐25 *M. oryzae* isolates. Analysis of variance (ANOVA) *p* values are shown above the boxplots. Different letters indicate significant differences among genotypes within an isolate based on Duncan's multiple range test (DMRT, *p* < 0.05). (e, f) Representative panicles of the *ko* and IR64 WT plants inoculated with (e) M64‐1‐3‐9‐1 and (f) IK81‐25. White bars denote 20 mm. PTC, premature termination codon in the mutants.

To evaluate the role of *Ptr_a_* in NB resistance, we generated knockout mutants in IR64, an NB‐resistant variety harboring *Ptr_a_*, using CRISPR‐Cas9‐mediated gene editing. A single guide RNA was designed to target the third exon of the *Ptr_a_* open reading frame (ORF) (Figure 4a). Five independent and homozygous T_2_ lines (*ko‐1* to *ko‐5*) with predicted loss‐of‐function mutations were selected and subjected to phenotypic analysis. Mutations in *ko‐1*, *ko‐2*, *ko‐3*, and *ko‐4* consisted of deletions of 49, 11, 5, and 6 bp, respectively, whereas *ko‐5* carried a single‐nucleotide C insertion (Figure 4b). Based on protein structure prediction, all mutations resulted in frameshifts and premature stop codons, disrupting the *Ptr_a_* ORF.

Phenotypic evaluation was performed by inoculating the knockout lines and wild‐type (WT) IR64 plants with two *M. oryzae* isolates, M64‐1‐3‐9‐1 and IK81‐25, both of which are avirulent on *Ptr_a_*‐containing lines (Table S9). All knockout (KO) lines developed typical NB symptoms, with average lesion lengths ranging from 40 to 110 mm and 50 to 110 mm for M64‐1‐3‐9‐1 and IK81‐25, respectively (Figure 4c,d). IR64 WT plants showed no visible lesions under the same conditions (Figure 4e,f). To assess potential off‐target effects, we sequenced the predicted off‐target region (Chr11:11,581,029), which was intact in all five independent KO lines (Figure S5), suggesting that the observed susceptibility resulted from *Ptr_a_* disruption rather than off‐target edits or T‐DNA insertions. These results indicate that *Ptr_a_* contributes to IR64 NB resistance against both isolates.

**FIGURE 5:**
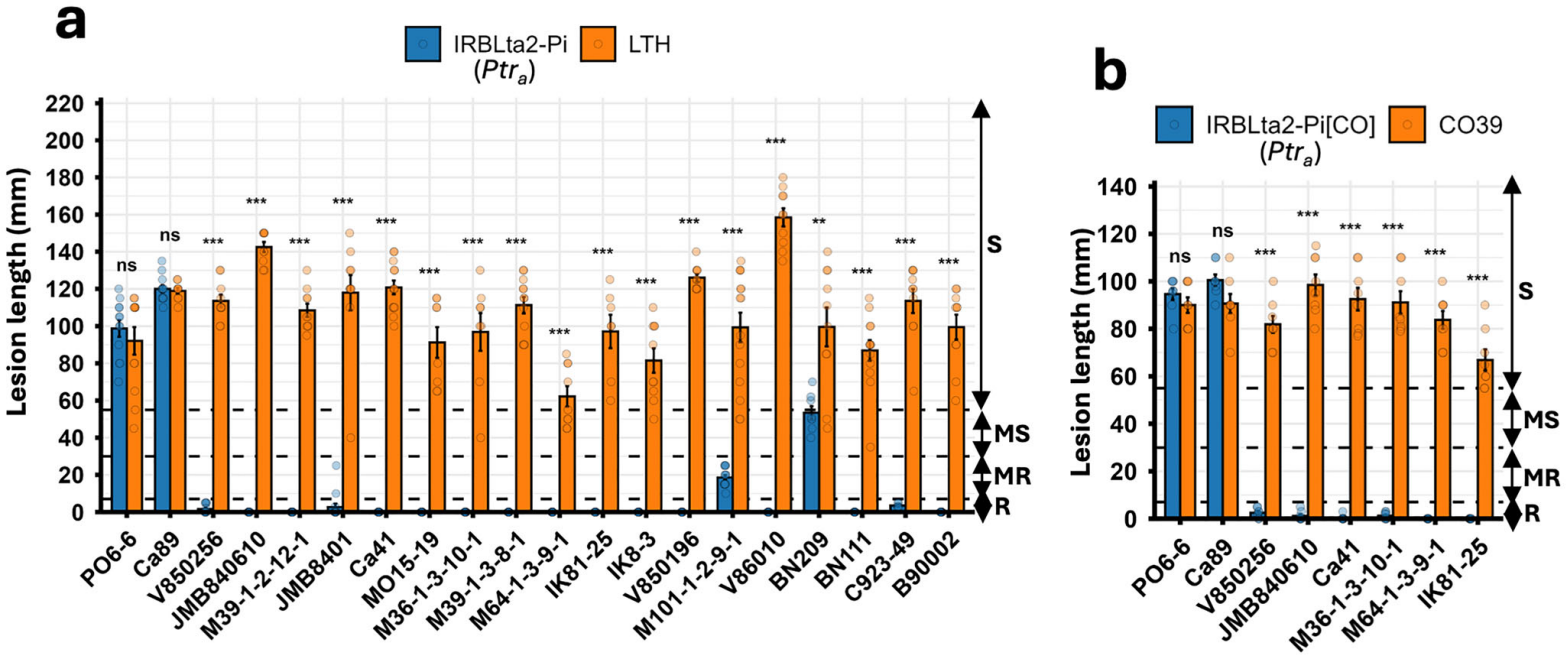
Neck blast reactions of *Ptr_a_* introgression lines and their recurrent parents. (a) IRBLta2‐Pi and Lijiangxintuanheigu (LTH; RP). (b) IRBLta2‐Pi[CO] and CO39 (RP). Bars show standard deviation, and overlaid points are individual observations. Dashed horizontal lines at 7, 30, and 55 mm mark the lesion‐length class boundaries: R (≤7 mm), MR (>7–30 mm), MS (>30–55 mm), and S (>55 mm). Within each isolate, significant differences between genotypes are indicated by asterisks (two‐tailed *t*‐test, *p < 0.05; **p < 0.01; ***p < 0.001; ns = not significant).

To examine the NB resistance spectrum conferred by *Ptr_a_*, we evaluated an LTH monogenic line (IRBLta2‐Pi) and a CO39 near‐isogenic line (IRBLta2‐Pi[CO]) both harboring the Pi No. 4‐derived *Ptr_a_* allele (Tsunematsu et al., 2000). Twenty differential blast isolates representing the Philippine *M. oryzae* diversity were inoculated onto IRBLta2‐Pi, while eight representative isolates were tested on IRBLta2‐Pi[CO]. We found that IRBLta2‐Pi was MR to resistant (R) against 17 out of 20 isolates (85%) (Figure 5a; Table S10), whereas *Ptr_a_* introgression in CO39 (IRBLta2‐Pi[CO]) conferred resistance to six of the eight isolates tested (75%) (Figure 5b; Table S10). These results indicate that *Ptr_a_* confers broad‐spectrum resistance to NB caused by the differential isolates from the Philippines. Furthermore, IRBLta2‐Pi also provided resistance against 16 leaf blast isolates (80%) (Table S10), demonstrating the role of *Ptr_a_* in both leaf blast and NB resistance.

*Pi‐ta*, a known leaf blast resistance gene, is located in the same locus as *Ptr_a_*. Therefore, we assessed the LTH monogenic line carrying *Pi‐ta* (IRBLta‐CP1) to distinguish the individual contributions of *Pi‐ta* and *Ptr_a_*. IRBLta‐CP1 was resistant to only four of the 20 isolates (20%), suggesting that *Pi‐ta* is not the major NB resistance gene (Table S9).

### 3.5 *Ptr_a_* is induced in neck tissues upon pathogen infection and activates pathogenesis‐related (PR) genes

**FIGURE 6:**
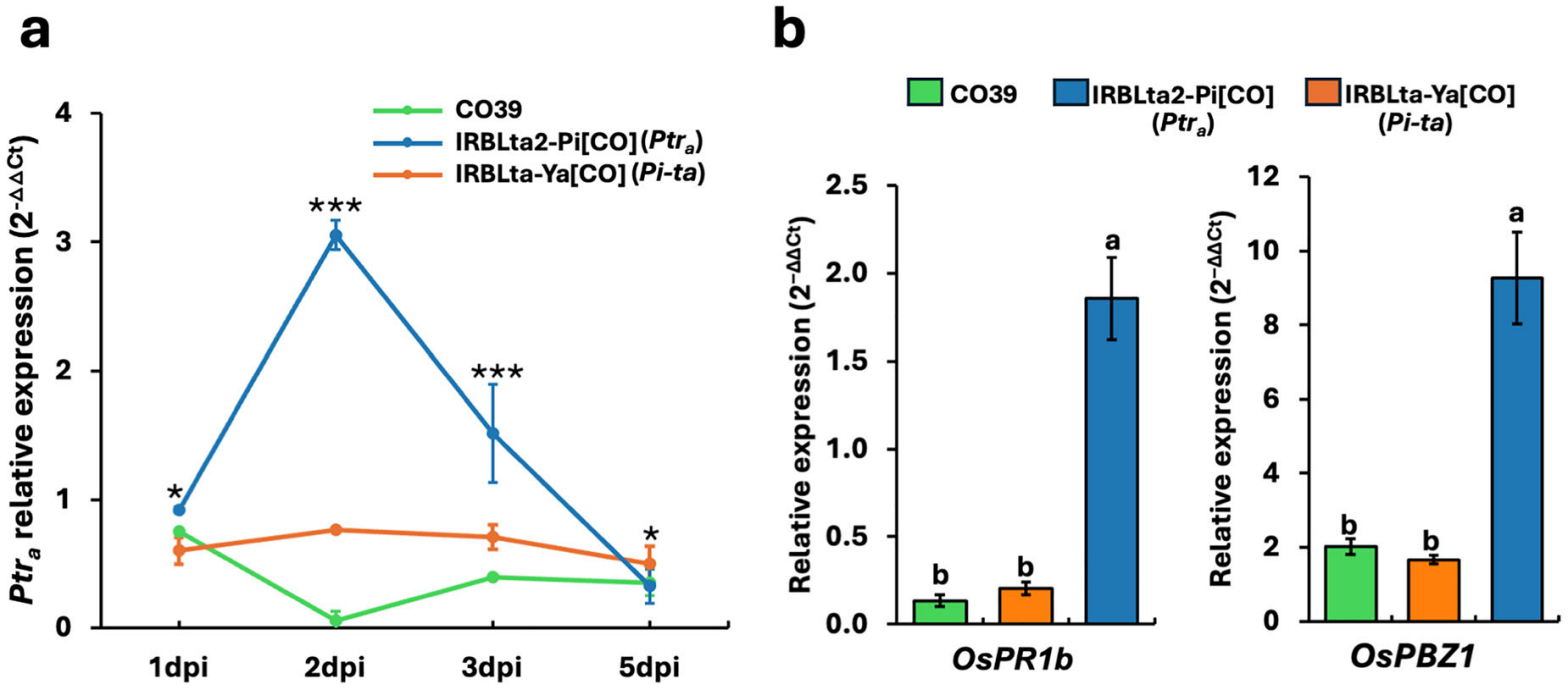
Relative expression levels of *Ptr_a_* and pathogenesis‐related genes upon neck blast infection. (a) Relative transcript abundance of *Ptr_a_* in CO39, IRBLta2‐Pi[CO], and IRBLta‐Ya[CO] at 1, 2, 3, and 5 days post‐inoculation (dpi). Relative mRNA level was quantified by quantitative reverse transcription polymerase chain reaction (qRT‐PCR) using *OsActin1* as the internal reference. Asterisks indicate statistically significant differences among group means (DMRT, *p < 0.05, **p < 0.01, ***p < 0.001). (b) Relative expression of pathogenesis‐related marker genes *OsPR1b* and *OsPBZ1* at 2 dpi. Bars represent mean ± SE of five biological replicates. Different letters above bars denote significant differences (DMRT, *p* < 0.05).

To dissect the *Ptr‐*mediated NB resistance mechanism, we assessed the expression dynamics of *Ptr_a_* upon pathogen infection in neck tissues. qRT‐PCR was performed on M64‐1‐3‐9‐1 and mock‐inoculated CO39 and NILs harboring *Ptr_a_* (IRBLta2‐Pi[CO]) and *Pi‐ta* (IRBLta‐Ya[CO]). We found that the relative expression of *Ptr_a_* was strongly induced in IRBLta2‐Pi[CO], peaking at 2 dpi with an approximately threefold increase relative to that in susceptible CO39 and IRBLta‐Ya[CO] lines, before declining by 5 dpi (Figure 6a). In contrast, the relative expression of *Ptr_a_* in IRBLta‐Ya[CO] was not significantly altered compared to that of CO39. These results indicate that infection by M64‐1‐3‐9‐1 induces *Ptr_a_* in the *Ptr_a_*‐carrying NIL, IRBLta2‐Pi[CO], but not in the *Pi‐ta*‐carrying NIL, IRBLta‐Ya[CO].

To examine the downstream responses of PR genes, we quantified the transcript levels of two marker genes for effector‐triggered immunity, *OsPR1b* (salicylic acid‐mediated defense and systemic acquired resistance) and *OsPBZ1* (probenazole‐inducible gene 1; marker of broad‐spectrum resistance). Both genes were significantly upregulated in IRBLta2‐Pi[CO] at 2 dpi but not in CO39 and IRBLta‐Ya[CO] (DMRT, *p* < 0.05) (Figure 6b). The increased expression of *Ptr_a_* and downstream PR genes upon pathogen inoculation supports the role of *Ptr_a_* as an active resistance gene that triggers defense responses.

### 3.6 Superior haplotype for neck blast resistance is rare in the 3K‐RGP but enriched in IRRI breeding lines

**FIGURE 7:**
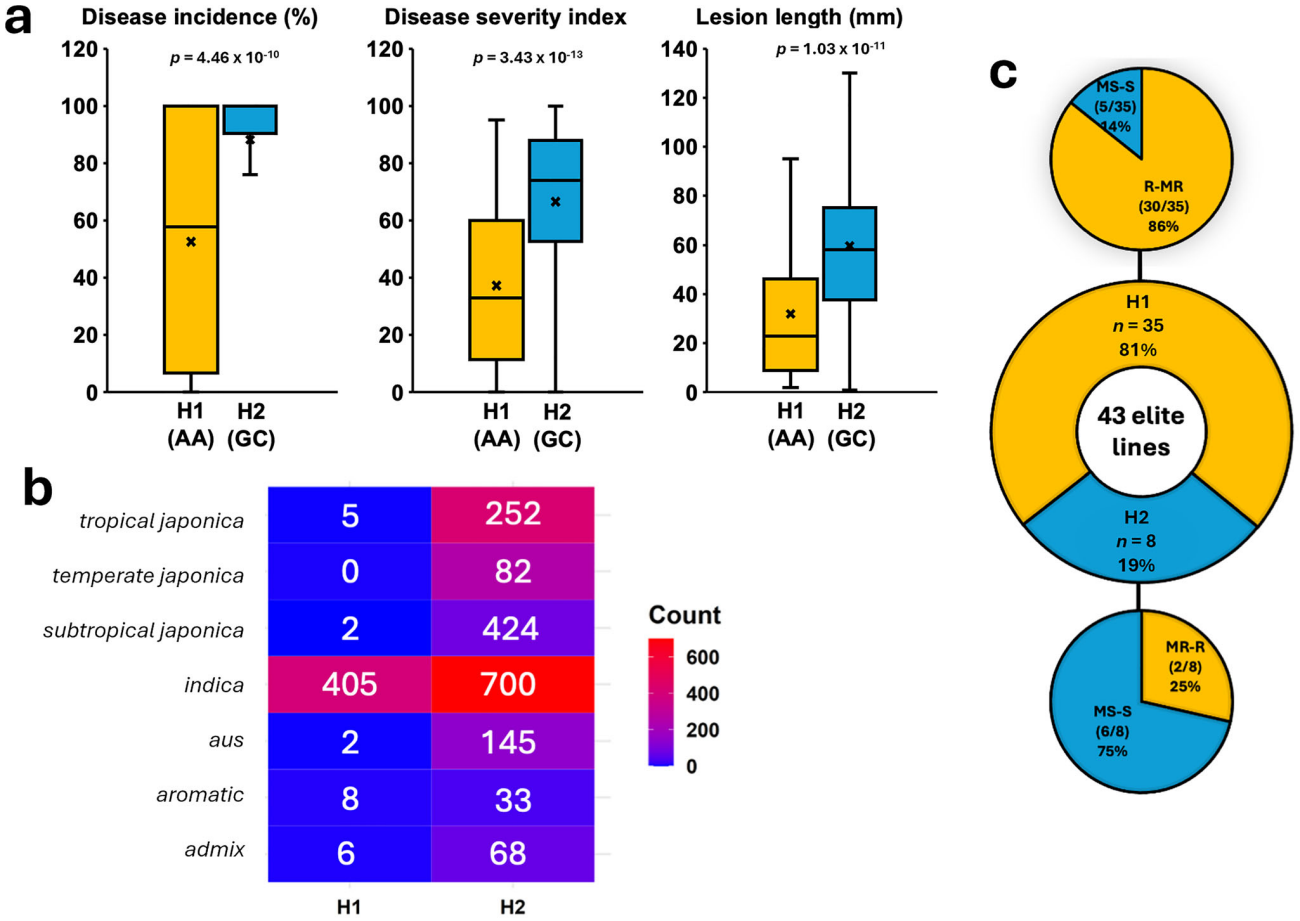
Haplotypes distribution across rice subpopulations and their association with neck blast resistance against M64‐1‐3‐9‐1 isolate. (a) Comparison of disease incidence, disease severity index, and lesion length between the two haplotypes H1 and H2. X represents the mean value. (b) Distribution of haplotypes (H1 and H2) across rice subpopulations. (c) Neck blast reactions and haplotype abundance in the International Rice Research Institute (IRRI) elite *indica* breeding lines classified as resistant (R, ≤7 mm), moderately resistant (MR, >7–30 mm), moderately susceptible (MS, >30–55 mm), and susceptible (S, >55 mm) based on mean lesion length. Absolute numbers and percentages of lines per group are indicated.

In the DI haploblock, two SNPs at Chr12:10,833,400 and Chr12:10,845,095 exceeded the Bonferroni‐corrected significance threshold for DI, DSI, and LL, explaining 15%/18%/14% and 15%/18%/13% of the phenotypic variance, respectively (Figure 2c, Table S7). As these two SNPs represented shared association signals within the same DI haploblock, they were used as reference SNPs for superior haplotype identification. Based on the allelic combinations at Chr12:10,833,400 and Chr12:10,845,095, the accessions were grouped into two haplotypes: H1 (AA) and H2 (GC). Among the 324 GWAS accessions with available information for the two reference SNPs, 82 and 242 accessions belonged to H1 and H2, respectively (Table 1; Table S1). H1 was associated with resistance, with significantly lower mean values for all disease components, including DI, DSI, and LL compared to H2 (Figure 7a); therefore, H1 was identified as a superior haplotype associated with NB resistance.

**TABLE 1:** Haplotype analysis based on two single nucleotide polymorphisms (SNPs), Chr12:10,833,400 and Chr12:10,845,095, in the 3K Rice Genome Panel (RGP) and International Rice Research Institute (IRRI) elite indica breeding lines.

Hap	Accession numbers in GWAS panel	Chr12: 10,833,400	Chr12: 10,845,095	DI (%)	DSI	LL (mm)	Freq. in the 3K RGP	Freq. in IRRI elites
H1	82	A	A	53.2^***^	38.7^***^	32.0^***^	0.20	0.83
H2	242	G	C	88.5	68.2	59.6	0.80	0.17

*Note*: Accessions were grouped into two haplotypes, H1 (AA) and H2 (GC), according to the allelic combinations at the two positions: Chr12:10,833,400 and Chr12:10,845,095.

Abbreviations: DI, disease incidence; DSI, disease severity index; Freq., frequency of accessions; GWAS, genome‐wide association study; Hap, haplotype; LL, lesion length.

***Significant at the 0.001 probability level.

To assess the breeding relevance and identify SNP markers for selecting NB‐resistant lines, we examined the distribution of the superior haplotype H1 among 2132 accessions from the 3K‐RGP GenBank and 168 IRRI elite *indica* breeding lines. Filtering SNPs from the 3K‐RGP classified the accessions into two groups, with 20% and 80% carrying H1 and H2, respectively (Table 1), indicating that the superior haplotype is relatively rare among GenBank accessions. At the subpopulation level, the superior haplotype H1 was abundant in the *indica* group (37%) but was absent from the *temperate japonica* group (Figure 7b; Table S5). Among accessions carrying H1, the proportion of resistant or moderately resistant reactions varied across genetic backgrounds (Table S1). In *indica*, 35 of 72 H1 accessions showed resistant or moderately resistant reactions. In *subtropical japonica*, all six H1 accessions exhibited resistant reactions. Concordance between H1 and resistance was also observed in *admix* (2/2) and *aromatic* (2/2) accessions, although the sample sizes in these groups were small. No accessions carrying H1 were identified in the *aus*, *temperate japonica*, or *tropical japonica* subpopulations in our GWAS panel; therefore, marker prediction accuracy could not be evaluated for these genetic backgrounds. Among the 168 IRRI elite *indica* breeding lines, 101 had complete genotype data for the two reference SNPs and were classified accordingly (Table S2). Of these, 84 lines (83%) carried the superior haplotype H1, indicating the abundance of the resistant haplotype in IRRI elite *indica* germplasm.

To test whether H1 could serve as a selection marker for NB resistance, we evaluated a random subset of 43 elite lines (Table S2, lines 1–43) against the isolate M64‐1‐3‐9‐1. Among them, 35 lines carried H1, of which 30 lines (86%) were resistant to moderately resistant (R‐MR), while the remainder were moderately susceptible to susceptible (MS‐S). In contrast, H2 carriers were predominantly MS‐S (6 out of 8 or 75%), with only two lines (25%) classified as R‐MR. Consequently, lines carrying H1 were approximately 18 times more likely to be R‐MR than those carrying H2 (odds ratio ≈ 18.0; based on 30/5 vs. 2/6, events [R‐MR]/non‐events [MS‐S]) (Figure 7c). These results indicate that H1 can be used to select NB‐resistant genotypes with a positive predictive accuracy of 86%.

## 4 DISCUSSION

In this study, we investigated the genetic basis of NB resistance in rice and identified *Ptr_a_* as a major gene underlying the resistance locus *qNB1* on chromosome 12. The Lys879 variant located in the C‐terminus of *Ptr_a_* was strongly associated with NB resistance. We also identified a superior haplotype (H1), defined by the A allele at Chr12:10,833,400 and the A allele at Chr12:10,845,095. H1 accurately predicts NB resistance with an accuracy of 86% and is prevalent among IRRI elite *indica* breeding lines. Consistent with earlier reports of the role of *Ptr_a_* in broad‐spectrum resistance against leaf blast, we found that this gene also provides broad‐spectrum resistance to NB, highlighting its potential in improving multi‐stage blast resistance in rice.

Through a GWAS using 335 rice accessions, we showed that the major locus for NB resistance maps to a 2.5‐Mb region (*qNB1*) on chromosome 12 harboring the *Ptr* gene. This association colocalized with the *Ptr* locus previously reported for panicle blast in a pan‐genome‐wide association study where 414 rice accessions were evaluated through natural field infection (McCouch et al., 2016; J. Wang et al., 2024). This suggests that the *Ptr* locus can modulate response to both panicle and NB. A significant association for leaf blast resistance was also detected for the *Ptr* gene (Greenwood et al., 2024), further demonstrating that *Ptr*‐linked resistance spans multiple blast infection stages. In this study, the ML harboring *Ptr_a_* (IRBLta2‐Pi) exhibited resistance against 17 and 16 NB and leaf blast isolates, respectively (Table S10). Such cross‐stage resistance is advantageous for breeding, as a single allele can enhance protection across leaf, panicle, and neck tissues.

Interestingly, *Ptr_a_* conferred resistance to NB but not to leaf blast caused by the isolate V86010 (Table S10), indicating that seedling‐stage responses do not necessarily predict resistance at later developmental stages. Although *Pi* genes are typically assessed using leaf assays, their effects may be stage‐ or tissue‐specific, with some defense responses expressed only in adult plants or in reproductive tissues (Du et al., 2024; Kalia & Rathour, 2019; Koyama et al., 2025). The contrasting responses observed between leaf blast and NB using the isolate V86010 further suggest that distinct defense mechanisms possibly operate in vegetative and reproductive tissues. Panicles and neck tissues undergo substantial physiological and transcriptional reprogramming during the reproductive stage (L. Wang et al., 2010) and may deploy specialized immune pathways that differ from those functioning in leaves. Accordingly, accessions that are susceptible to leaf blast at the seedling stage may still exhibit resistance to NB at the reproductive stage. These findings underscore the importance of evaluating blast resistance across multiple developmental stages and support the notion that NB resistance represents a genetically and physiologically distinct component of the rice blast resistance spectrum. Standard differential rice lines, including the LTH monogenic lines and CO39 NILs carrying single leaf blast resistance genes, have been widely used for leaf blast evaluation (Tsunematsu et al., 2000). Testing these lines against NB with diverse isolates will clarify whether leaf blast resistance genes also confer protection at the reproductive stage.

The tight linkage between *Pi‐ta* and *Ptr* has historically complicated genetic dissection because both genes are located in a low‐recombination region near the chromosome 12 centromere (J. Wu et al., 2003). Although originally thought to be allelic (Rybka et al., 1997), subsequent analyses established that *Ptr* is distinct from *Pi‐ta* (Biswas et al., 2023; Meng et al., 2020; Zhao et al., 2018). *Pi‐ta* encodes a nucleotide‐binding site‐leucine‐rich repeat (NBS‐LRR) protein (Bryan et al., 2000), whereas *Ptr* encodes an ARM protein that confers broad‐spectrum resistance against leaf blast (Zhao et al., 2018). Although closely located, the two genes act independently in mediating blast resistance (Meng et al., 2020; G. Xiao et al., 2024). In our results, *Ptr_a_* conferred broader‐spectrum resistance compared with *Pi‐ta* to both leaf and NB. The *Ptr_a_*‐harboring monogenic line, IRBLta2‐Pi, was resistant to 17 of the 20 differential blast isolates from the Philippines, while the *Pi‐ta*‐harboring IRBLta‐CP1 line was resistant to only four (Table S9). These results support the conclusion that *Ptr_a_* is a major contributor to NB resistance but not *Pi‐ta*.

Unlike the classical nucleotide‐binding leucine‐rich repeat (NLR) genes, which often exhibit extensive allelic diversification due to strong selection pressures and frequent presence/absence polymorphisms (Yang et al., 2008), several non‐NLR resistance genes such as *Xa4* and *Xa21* in rice present comparatively limited natural variation (Edirisinghe et al., 2024; Nanayakkara et al., 2018). Unlike other non‐NLR genes, *Ptr* possesses several allelic variants including *Ptr_a_*, *Ptr_b_*, *Ptr_c_*, *Ptr_d_*, and *Ptr_e_*, which are distinguished by amino acid substitutions and small Indels in their C‐terminus, specifically in the last 50 residues downstream of the ARM domain. Among these alleles, *Ptr_a_* lacks four residues at positions 870–873 (lysine‐proline‐glutamate‐lysine) in its C‐terminus but can recognize 11 known natural variants of *AVR‐Pita*, making it a broad‐spectrum leaf blast resistance gene (Greenwood et al., 2024; G. Xiao et al., 2024). Here, we identified a C‐terminus Lys879 variant located downstream of the ARM domain and was consistently found among accessions harboring *Ptr_a_* and *Ptr_c_*. This Lys879 variant was associated with NB resistance (Figure 3), suggesting that this variant may serve as a reliable marker for detecting *Ptr_a_*‐ and *Ptr_c_*‐ associated NB resistance.

Although the C‐terminus of *Ptr* appears critical for resistance function, its mechanistic role in effector recognition remains unresolved. A recent study revealed that *AVR‐Pita* is the cognate effector of *Ptr* (G. Xiao et al., 2024). Because *Ptr_a_* and *Ptr_c_*, which carry the Lys879 variant at the C‐terminus, are associated with NB resistance, we hypothesize that this residue influences the *AVR‐Pita* binding affinity or recognition specificity. Future experiments, such as yeast two‐hybrid assays, co‐immunoprecipitation, structural analyses (e.g., cryo‐EM), will be necessary to test this hypothesis. Understanding how *Ptr_a_* recognizes *AVR‐Pita* will be critical for predicting resistance durability, given the rapid evolutionary turnover of *M. oryzae* effector repertoires (Chuma et al., 2011; Dai et al., 2010).

The *Ptr_a_* allele is expected to be effective across blast populations harboring *AVR‐Pita* because it recognizes all 11 known natural variants of this effector (Greenwood et al., 2024; G. Xiao et al., 2024). Studies from southern China, Vietnam, the Philippines, and southern India indicate that *AVR‐Pita* occurs at relatively high frequencies at 72%, 58%, 57%, and 47%, respectively (Amoghavarsha et al., 2022; X. Chen et al., 2025; Lopez et al., 2019; Trang et al., 2019). This indicates the value of deploying *Ptr_a_* in these countries. Continued monitoring of *M. oryzae* populations is essential for targeted deployment. Coordinated evaluations across countries such as those led by the Global BioNET network established by the IRRI would provide standardized data to guide durable deployment strategies and reduce the risk of resistance breakdown. Here, although we showed that *Ptr_a_* conferred broad‐spectrum NB resistance in multiple genetic backgrounds, including CO39, LTH, IR64, and selected IRRI elite *indica* breeding lines, validation across a wider set of varieties is needed. In addition, although the MLs and NILs displayed resistant reactions across the majority of differential blast isolates from the Philippines, the performance of *Ptr_a_* should also be evaluated across a broader regional panel of isolates before a large‐scale deployment.

In the GWAS panel, only 20% of the accessions carried the superior H1 haplotype, indicating that it remains relatively rare. Subpopulation differences in NB response were evident: *aus* and *tropical japonica* showed the highest susceptibility, followed by *indica* as moderately susceptible, *temperate japonica* as moderate resistant and *subtropical japonica* as highly resistant. These subpopulation differences are consistent with the previous studies on leaf and panicle blast (Fan et al., 2024; Volante et al., 2020; J. Wang et al., 2024; Yu et al., 2022). Although H1 occurs frequently in *indica*, the overall NB resistance in this group remains low because *Ptr* explains a modest 14%–18% of the phenotypic variance in a diverse panel. Additional loci within *indica* likely contribute to susceptibility. Conversely, in *subtropical* and *temperate japonica* accessions, NB resistance was observed despite the low frequency of the superior haplotype, implying the presence of other resistance loci that may compensate for the absence of *Ptr_a_*. Population‐specific analyses will be necessary to uncover these additional determinants of NB resistance.

The superior haplotype H1 was already enriched in elite *indica* breeding lines, providing an immediate resource for MAS. In these lines, the high predictive accuracy (∼86%) of H1 likely reflects their more uniform genetic background, where locus effects are more consistent. The high frequency of H1 in these lines suggests that breeders may have unintentionally selected for this haplotype during past improvement cycles. Such enrichment illustrates a broader pattern in rice breeding, where selection for agronomic traits can inadvertently alter the frequency of resistance alleles. For example, intensive selection for yield and quality traits reduced the prevalence of the *Piz*‐*t* allele in some breeding groups (Thakur et al., 2013; N. Xiao et al., 2021). Similarly, analysis of NLR repertoires in rice detected signatures of balancing and positive selection on blast‐related immune receptors such as RGA4 in *indica* landraces, consistent with long‐term selection maintaining resistance specificities (Gladieux et al., 2024). In our GenBank accessions and the *indica* breeding lines, H1 alone does not fully explain the phenotypic variation and lines carrying H2 also showed resistant phenotypes, indicating that there are complementary loci contributing to NB resistance. Thus, while H1 and key SNP markers (Table S7) are useful markers for selecting NB resistance, a multi‐locus breeding strategy that integrates *Ptr_a_* with additional QTLs or incorporates genomic selection will benefit durable resistance. This is important because resistance conferred by single R genes can be rapidly overcome by pathogen evolution (Lore et al., 2025).

Although our validation confirmed that *Ptr_a_* is required for broad‐spectrum resistance against the 17 differential blast isolates from the Philippines, the genetic architecture of NB resistance is possibly more complex. Several association signals surpassed the Bonferroni significance threshold (Table S6), indicating that additional loci may influence the phenotype. Genes harbored in the *qNB1* locus may harbor variants that enhance or modify NB resistance together with *Ptr_a_*. Some defense‐related genes such as *Pi‐ta*, *Pi42(t)*, *OsPUB23*, *OsCYCA1*, *OsPR4*, and *OsPP130* were identified within the *qNB1* locus (Figure S6). These genes could function in parallel defense pathways, contribute to quantitative resistance, or modulate the expression or effectiveness of *Ptr_a_*. Functional characterization of these candidates is therefore needed to determine whether they act independently, additively, or epistatically with *Ptr_a_* in shaping NB resistance.

Defining the haploblock helps to clarify the genetic basis of resistance and provides SNP markers for marker‐assisted breeding. The 42.3‐kb haploblock identified contained 19 SNPs in strong linkage with the DI peak SNP, any of which could serve as diagnostic markers for NB resistance. However, the predictive strength of individual SNPs may differ depending on population structure and recombination history (Y. Zhang et al., 2023). Mapping resolution is similarly influenced by historical recombination and LD patterns in the germplasm used (Padmashree et al., 2023). In rice, haplotype‐based GWAS approaches can sometimes outperform single‐SNP analyses, emphasizing that not all variants within a haploblock have equal predictive value (Abed & Belzile, 2019). For breeding applications, prioritizing reference SNPs or a minimal set of highly informative markers will maximize efficiency while maintaining accuracy. The presence of multiple tightly linked signals also provides flexibility for marker design, allowing breeders to select SNPs compatible with their genotyping platforms without compromising reliability. Here, we identified two SNPs in the same LD or haploblock with DI peak SNP, which can be used as selection markers. This allows breeders to incorporate *Ptr*‐based resistance into their breeding programs.

In summary, our study identified *Ptr_a_* as the primary gene underlying broad‐spectrum resistance to neck and leaf blast caused by the differential blast isolates from the Philippines. The Lys879 variant and superior H1 haplotype identified serve as predictors of resistance across GenBank germplasms as well as elite *indica* breeding lines. The provided SNP markers are suitable for accelerating the breeding of blast‐resistant rice varieties. Beyond establishing the central role of *Ptr_a_*, our work illustrates the power of integrating genome‐wide association, allele mining, and genome editing to uncover resistance mechanisms. The characterization of an effective non‐NLR resistance gene highlights the contribution of noncanonical immune components and provides opportunities for precision breeding to increase disease resilience. Collectively, these insights provide a strong foundation for the strategic development of durable blast resistance in global rice improvement programs.

## AUTHOR CONTRIBUTIONS

**Ian Paul Navea**: Data curation; formal analysis; methodology; validation; visualization; writing—original draft; writing—review and editing. **Mohammad Abdul Monsur**: Data curation; formal analysis; investigation; methodology; validation; visualization; writing—original draft; writing—review and editing. **Mary Jeanie Telebanco‐Yanoria**: Data curation; formal analysis; investigation; methodology; resources; supervision; writing—review and editing. **Dianne Gene De La Rosa**: Data curation; formal analysis; methodology; validation; writing—review and editing. **Sherry Lou Hechanova**: Data curation; formal analysis; methodology; validation; writing—review and editing. **Arvin Paul Tuaño**: Data curation; formal analysis; investigation; methodology; validation; visualization; writing—review and editing. **Christian Joseph Cumagun**: Supervision; writing—review and editing. **Il‐Ryong Choi**: Funding acquisition; supervision; writing—review and editing. **Suresh Kadaru**: Conceptualization; resources; supervision; writing—review and editing. **Sung‐Ryul Kim**: Conceptualization; investigation; methodology; resources; supervision; validation; writing—review and editing. **Bo Zhou**: Conceptualization; funding acquisition; investigation; project administration; resources; supervision; writing—review and editing. **Van Schepler‐Luu**: Conceptualization; funding acquisition; investigation; methodology; project administration; resources; supervision; writing—original draft; writing—review and editing.

## CONFLICT OF INTEREST STATEMENT

The authors declare no conflicts of interest.

## Supporting information




**Table S1. Neck blast reactions of the 335 GWAS accessions inoculated with M64‐1‐3‐9‐1**. Values represent mean values of DI = disease incidence (%); DSI = disease severity index; LL = lesion length (mm).*Values represent lesion length (mm) and are classified as resistant (R, 0–7 mm), moderately resistant (MR, > 7‐30 mm), moderately susceptible (MS, > 30‐55 mm), or susceptible (S, > 55 mm).
**Table S2. Neck blast reactions of IRRI elite *indica* breeding lines inoculated with M64‐1‐3‐9‐1**. Values represent lesion length (mm) and are classified as resistant (R, 0–7 mm), moderately resistant (MR, > 7‐30 mm), moderately susceptible (MS, > 30‐55 mm), or susceptible (S, > 55 mm).
**Table S3. Modified evaluation scale for neck blast disease in IRRI Plant Pathology Laboratory** (Yanoria et al., 2025)
**Table S4. Primers used in the qRT‐PCR experiments and for potential off‐target sequencing in CRISPR‐Cas9 mutants**.
**Table S5. Subpopulation‐level neck blast reactions of the 335 GWAS accessions inoculated with M64‐1‐3‐9‐1**. DI = disease incidence (%); DSI = disease severity index; LL = lesion length (mm). *Values indicate number of accessions in the 3K RGP.
**Table S6. List of SNPs exceeding the Bonferroni significance threshold value ‐log_10_(*P*) = 6.7**.
**Table S7. List of SNPs located within the 42.3‐kb haploblock on chromosome 12 (Chr12:10,822,390‐Chr12:10,864,690)**. DI = disease incidence (%); DSI = disease severity index; LL = lesion length (mm).
**Table S8. Neck blast reactions of five *Ptr* variants in the GWAS accessions**. Values represent mean values of DI = disease incidence (%); DSI = disease severity index; LL = lesion length (mm). Genotype information of the A/G SNP at Chr. 12: 10,833,400 is provided for each Ptr alleles. Classification was based on lesion length and are classified as resistant (R, 0–7 mm), moderately resistant (MR, > 7‐30 mm), moderately susceptible (MS, > 30‐55 mm), or susceptible (S, > 55 mm).
**Table S9. Neck blast reactions of the *Ptr* and *Pi‐ta* 25 LTH monogenic lines inoculated with 20 differential blast isolates from the Philippines**. Values represent lesion length (mm) and are classified as resistant (R, 0–7 mm), moderately resistant (MR, > 7‐30 mm), moderately susceptible (MS, > 30‐55 mm), or susceptible (S, > 55 mm). LTH was included as susceptible check.
**Table S10. Neck and leaf blast reactions of the monogenic line harboring *Ptr_a_***. The line was inoculated with the 20 differential blast isolates from the Philippines. Values represent lesion length (mm) and are classified as resistant (R, 0–7 mm), moderately resistant (MR, > 7‐30 mm), moderately susceptible (MS, > 30‐55 mm), or susceptible (S, > 55 mm). LTH was included as susceptible check for neck and leaf blast. ND = No data.

**Figure S1. Repeatability of neck blast phenotypes across two independent experiments**. Pearson correlation between lesion length measurements obtained from two independent experiments using the modified inoculation method (Yanoria et al., 2025). The analysis included all measurements from the accessions used in the validation experiments that were repeated twice including the CRISPR‐Cas9 ko lines (5 genotypes x 2 isolates, *n* = 10) and WT/control check plants (4 genotypes x 2 isolates, *n* = 8).
**Figure s2. Assessment of phenotype normality prior to GWAS**. Quantile‐quantile (Q‐Q) plots showing the distribution of disease incidence (DI), disease severity index (DSI), and lesion length (LL). DI and DSI deviated from normality with Kolmogorov‐Smirnov test: *D* = 0.34, *p* < 0.001 and *D* = 0.12, *p* < 0.001, respectively and were therefore transformed using an arcsine square‐root transformation prior to GWAS analysis. LL approximated a normal distribution (*D* = 0.05, *p* = 0.31) and was analyzed without transformation.
**Figure S3. Map of the CRISPR‐Cas9 binary vector pSR339 (16,309 bp) used for *Ptr_a_* gene editing**. The T‐DNA region (between the right border [RB] and left border [LB]) contains three expression cassettes. (i) SpCas9, driven by the maize ubiquitin promoter (pZmUbi1) and terminated by the nopaline synthase terminator (tNOS). (ii) A guide RNA (sgRNA) cassette, in which the target‐specific sgRNA is inserted at *AarI* restriction sites by replacing the LacZ fragment; sgRNA expression is driven by the rice U3 promoter (pOsU3). (iii) The selectable marker hygromycin phosphotransferase (HPT), driven by the CaMV 35S promoter (p35S) and terminated by the 35S terminator (t35S).
**Figure S4. Melting curve analysis of qRT‐PCR primer pairs used in this study**.
**Figure S5. Off‐target analysis for gRNA targeting *Ptr_a_***. Sequence of potential off‐targets in five *Ptr_a_* knockout mutants at Chr11:11,581,029. IR64 WT was used as reference.
**Figure S6. Position of known defense‐related genes in *qNB1* on Chromosome 12**. Manhattan plot showing association signals across Chromosome 12, with the *qNB1* locus spanning approximately 2.5 Mb (Chr12:10,350,016‐Chr12:12,885,558). The red line denotes the Bonferroni significance threshold. The *qNB1* region contains known defense‐related genes, including *Ptr*, *Pi‐ta*, *Pi42(t)*, *OsPUB23*, *OsCYCA1*, *OsPR4*, and *OsPP130*.

## Data Availability

The data of this study are presented in the main tables, figures, and supplementary tables and figures. SNP data can be retrieved from https://snp‐seek.irri.org/. Supplementary tables and GWAS phenotypic data are available at https://doi.org/10.5281/zenodo.20025230.
