# Supplementary material for: Natural variation in the atypical resistance gene Ptr confers broad‐spectrum neck blast resistance in rice
Source: Plant Genome. 2026 Aug 12;19(3):e70290. doi: 10.1002/tpg2.70290 (PMC13492586; doi:10.1002/tpg2.70290)
Supplement: Supplementary file 1 [file TPG2-19-e70290-s001.pdf]

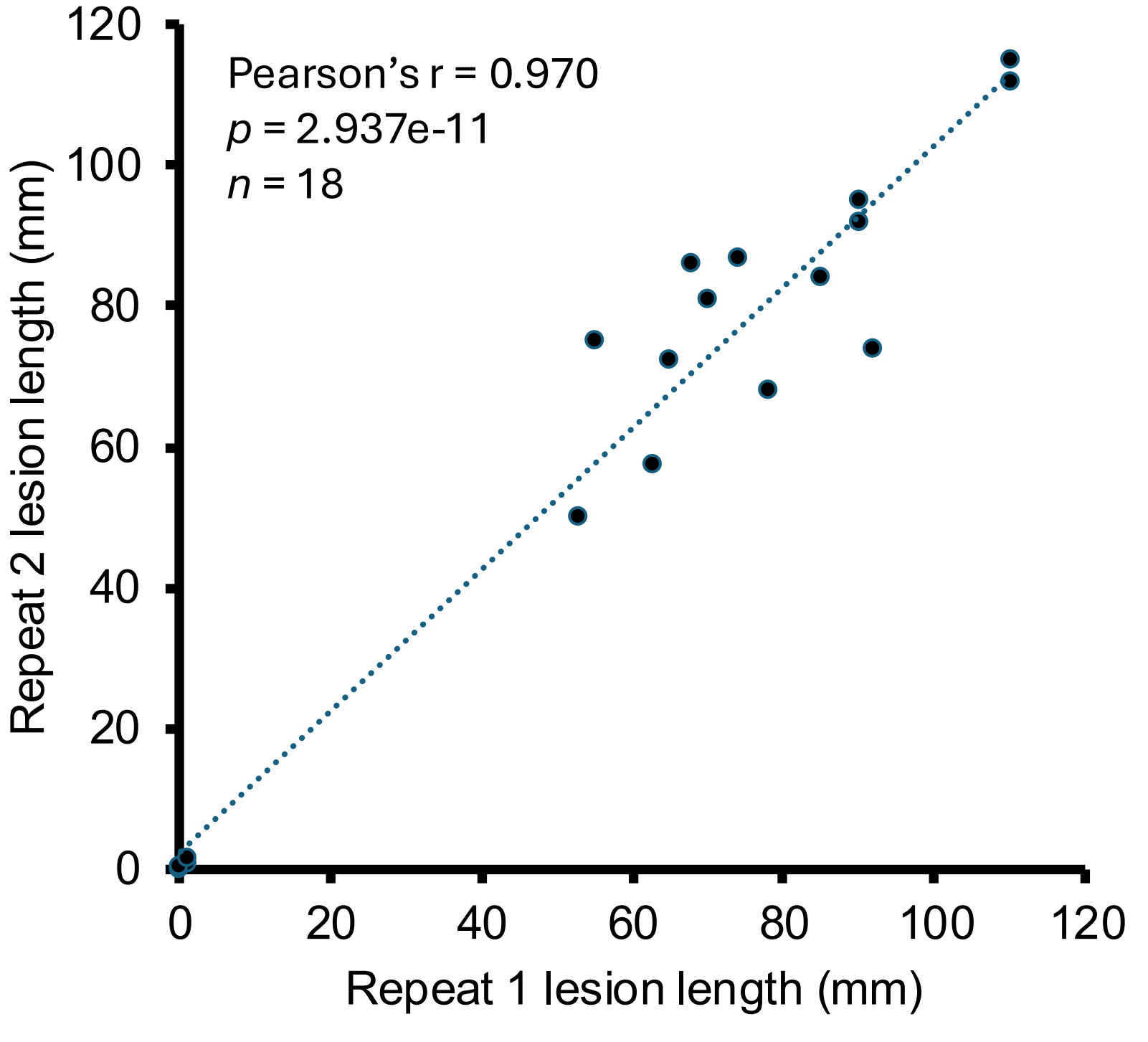

Figure S1. Repeatability of neck blast phenotypes across two independent experiments. Pearson correlation between lesion length measurements obtained from two independent experiments using the modified inoculation method (Yanoria et al., 2025). The analysis included all measurements from the accessions used in the validation experiments that were repeated twice including the CRISPR-Cas9 knockout lines (5 genotypes x 2 isolates, *n* = 10) and WT/control check plants (4 genotypes x 2 isolates, *n* = 8).

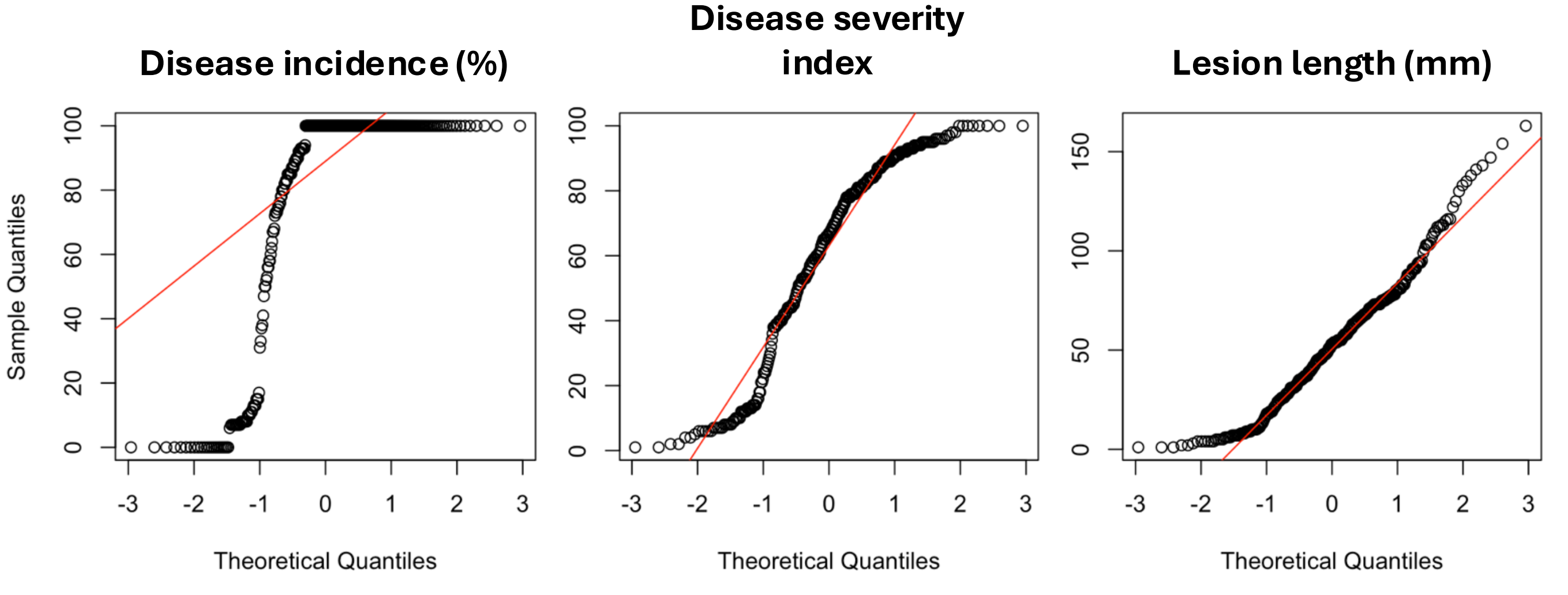

Figure S2. Assessment of phenotype normality prior to GWAS. Quantile-quantile (Q-Q) plots showing the distribution of disease incidence (DI), disease severity index (DSI), and lesion length (LL). DI and DSI deviated from normality with Kolmogorov-Smirnov test: $D = 0.34$, $p < 0.001$ and $D = 0.12$, $p < 0.001$, respectively and were therefore transformed using an arcsine square-root transformation prior to GWAS analysis. LL approximated a normal distribution ($D = 0.05$, $p = 0.31$) and was analyzed without transformation.

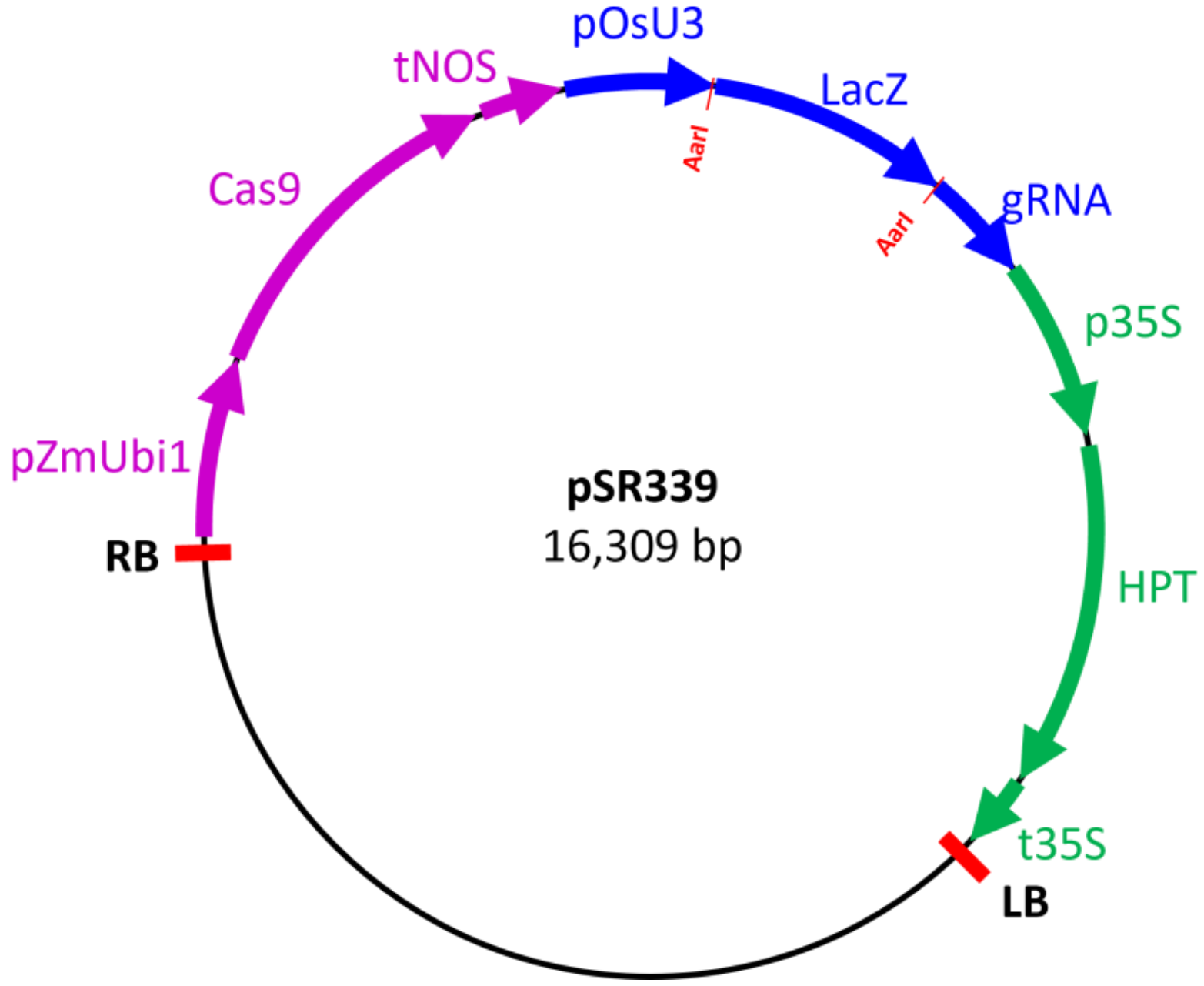

Figure S3. Map of the CRISPR-Cas9 binary vector pSR339 (16,309 bp) used for *Ptr*$_a$ gene editing. The T-DNA region (between the right border [RB] and left border [LB]) contains three expression cassettes. (i) SpCas9, driven by the maize ubiquitin promoter (pZmUbi1) and terminated by the nopaline synthase terminator (tNOS). (ii) A guide RNA (sgRNA) cassette, in which the target-specific sgRNA is inserted at *AarI* restriction sites by replacing the LacZ fragment; sgRNA expression is driven by the rice U3 promoter (pOsU3). (iii) The selectable marker hygromycin phosphotransferase (HPT), driven by the CaMV 35S promoter (p35S) and terminated by the 35S terminator (t35S).

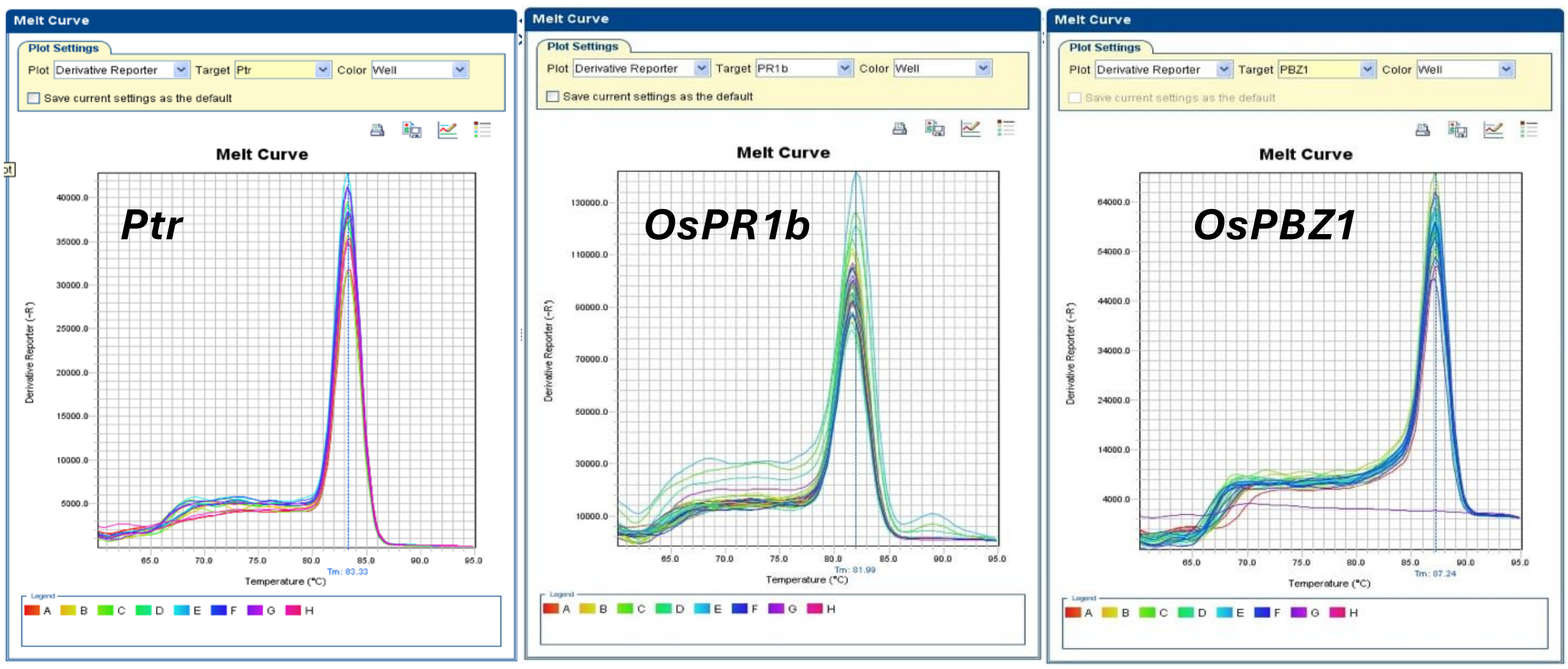

Figure S4. Melting curve analysis of qRT–PCR primer pairs used in this study.

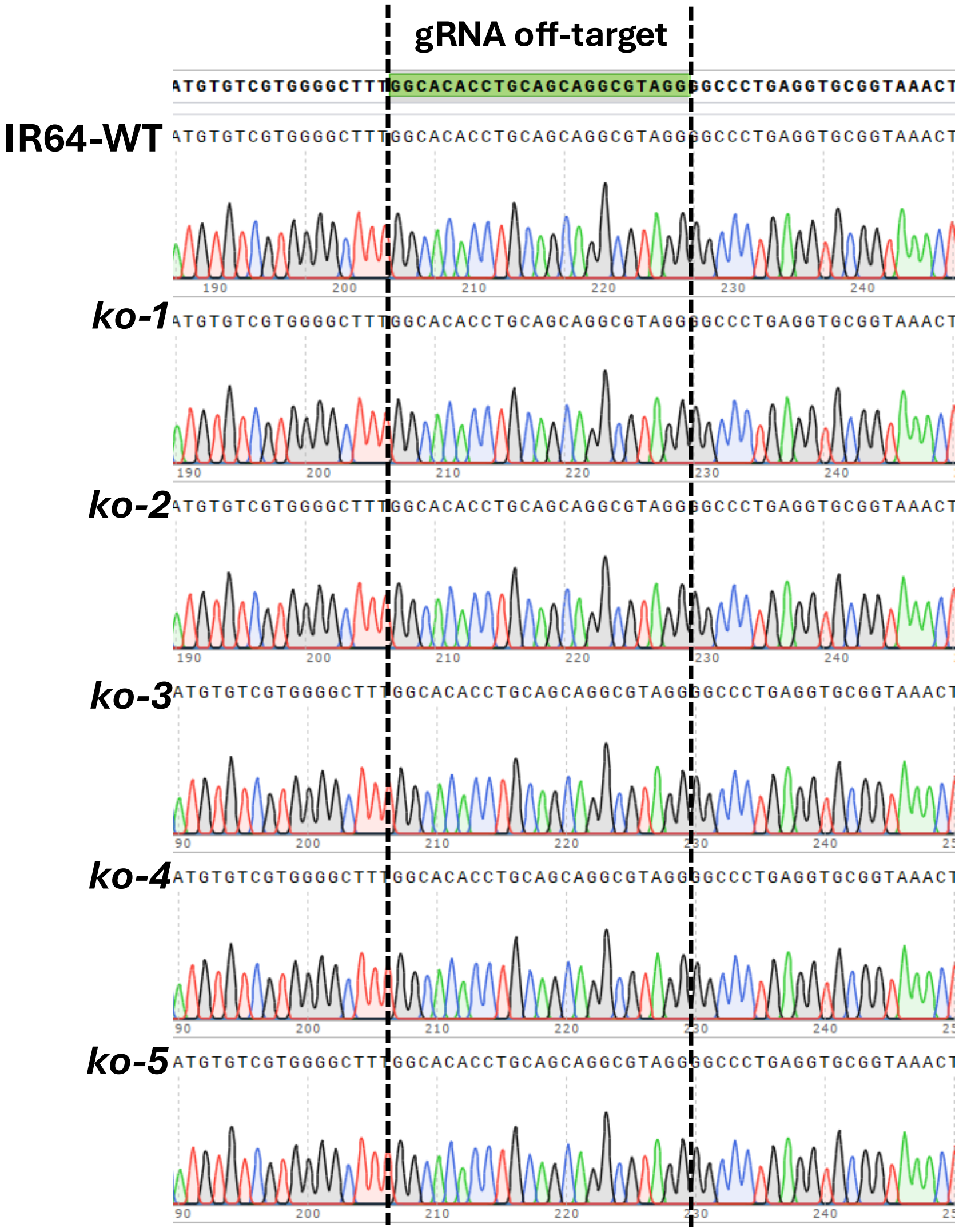

Figure S5. Off-target analysis for gRNA targeting *Ptr*$_a$. Sequence of potential off-targets in five *Ptr*$_a$ knockout mutants at Chr11:11,581,029. IR64 WT was used as reference.

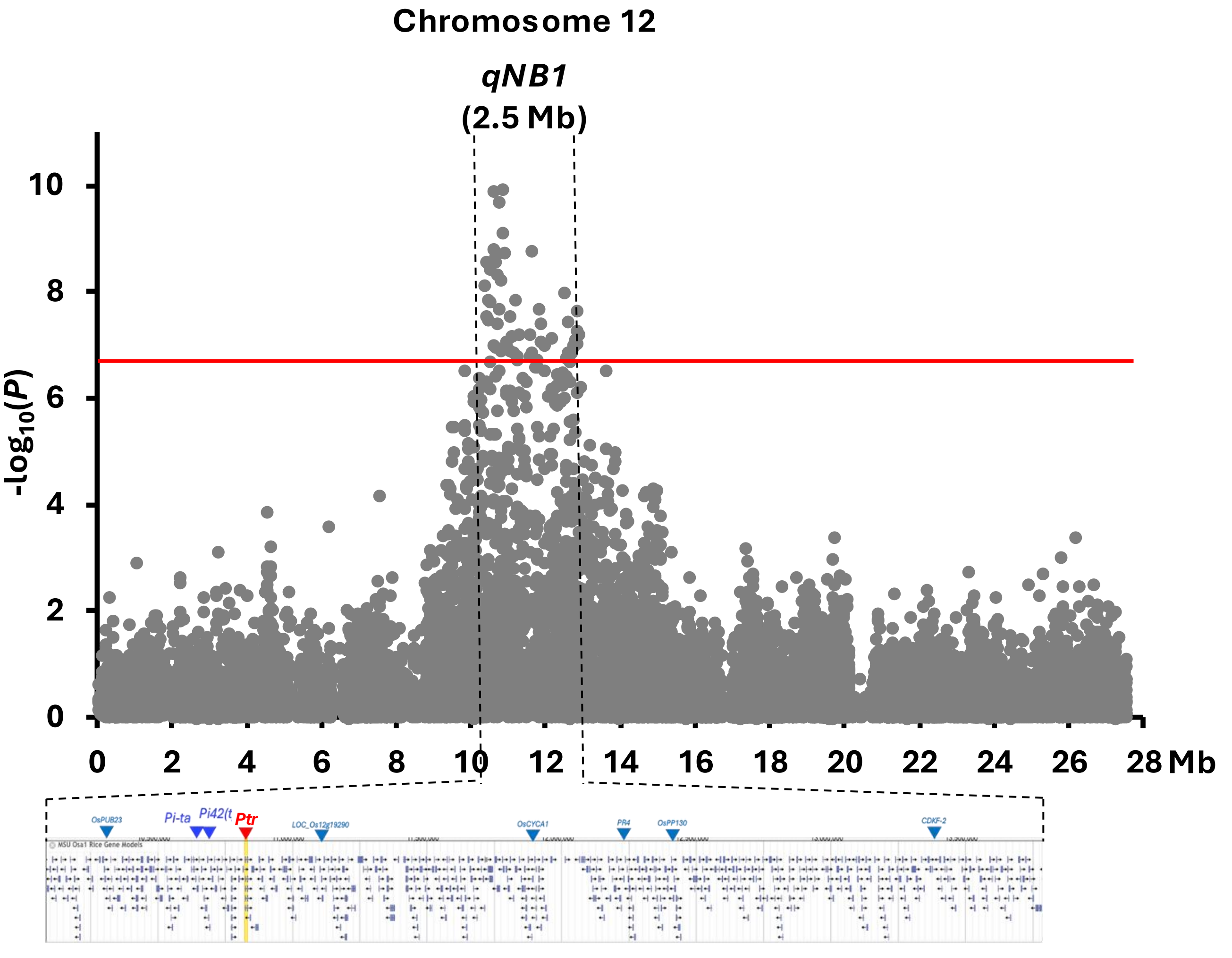

Figure S6. Position of known defense-related genes in *qNB1* on Chromosome 12. Manhattan plot showing association signals across Chromosome 12, with the qNB1 locus spanning approximately 2.5 Mb (Chr12:10,350,016-Chr12:12,885,558). The red line denotes the Bonferroni significance threshold. The *qNB1* region contains known defense-related genes, including *Ptr*, *Pi-ta*, *Pi42(t)*, *OsPUB23*, *OsCYCA1*, *OsPR4*, and *OsPP130*.
